# Effects of Balanced Dietary Patterns and/or Integrated Exercise on Serum 1,5-Anhydroglucitol and CVD Risk Factors in Individuals with Prediabetes

**DOI:** 10.3390/life16020198

**Published:** 2026-01-25

**Authors:** Ting Zhu, Da Pan, Lanlan Gui, Wei Yan, Ligang Yang, Wang Liao, Shaokang Wang, Guiju Sun

**Affiliations:** 1 Key Laboratory of Environmental Medicine and Engineering of Ministry of Education, Department of Nutrition and Food Hygiene, School of Public Health, Southeast University, Nanjing 210009, China; zhutingls@163.com (T.Z.); pantianqi92@foxmail.com (D.P.); guilanlan970713@163.com (L.G.); yan03260326@163.com (W.Y.); yangligang2012@163.com (L.Y.); wangliao@seu.edu.cn (W.L.); gjsun@seu.edu.cn (G.S.); 2 Clinical Medical Research Center for Plateau Gastroenterological Disease of Xizang Autonomous Region, School of Medicine, Xizang Minzu University, Xianyang 712082, China

**Keywords:** 1,5-anhydroglucitol (1,5-AG), cardiovascular disease (CVD), balanced dietary, integrated exercise, prediabetes, diabetes mellitus (DM)

## Abstract

This study aimed to identify metabolomic biomarkers for diabetes progression and validate their response to lifestyle intervention. A two-phase design was employed: first, untargeted metabolomics distinguished normoglycemic, prediabetic (PDM), and diabetic (DM) individuals, identifying 1,5-anhydroglucitol (1,5-AG) as the most significant biomarker for differentiating PDM from DM (apparent AUC = 0.97, 95% CI: 0.95–1.00; corrected AUC = 0.94, 95% CI: 0.83–1.00; *q* < 0.001). Second, in a 3-month randomized controlled trial involving 300 adults with PDM, the combined diet and exercise intervention significantly improved fasting blood glucose and glycated hemoglobin levels, while concurrently elevating serum 1,5-AG levels compared with the control group, though it did not yield significant improvements in other cardiovascular disease-related risk factors including body mass index, waist circumference, systolic blood pressure, and diastolic blood pressure. The intervention also showed a trend toward reduced diabetes incidence. Integrated analysis establishes 1,5-AG as a sensitive biomarker of dysglycemia that is responsive to lifestyle modification, supporting its potential as a mechanistic tool for monitoring intervention efficacy in diabetes prevention.

## 1. Introduction

Prediabetes (PDM) generally refers to a metabolic state in which blood glucose levels are above the normal range but below the diagnostic threshold for diabetes [1,2]. It portrays a high-risk state for the future development of diabetes mellitus (DM) and cardiovascular disease (CVD) [3,4,5,6], particularly among middle-aged and older adults [7,8]. With its escalating global prevalence, implementing effective interventions during this reversible stage has become a crucial public health strategy. Lifestyle modification, particularly through diet and exercise, remains the cornerstone for preventing diabetes progression.

Untargeted metabolomics provides a powerful tool for identifying novel biomarkers capable of differentiating between normoglycemia, PDM, and DM [9,10,11,12,13,14]. Serum 1,5-anhydroglucitol (1,5-AG) is one such marker, utilized in clinical practice for monitoring postprandial glucose variations in DM. It has been linked to serious complications, including end-stage renal disease, subclinical myocardial injury, cardiovascular events, and all-cause mortality [15,16,17]. Despite this established role, its utility as a biomarker for stratifying individuals across the entire glycemic spectrum and specifically for identifying and characterizing the metabolically vulnerable prediabetes state awaits further validation. In particular, systematic comparisons simultaneously evaluating its discriminatory power among normoglycemic, prediabetic and diabetic individuals within the same cohort are sparse, and its response to preventive lifestyle interventions in the prediabetic stage remains largely unexplored.

Concurrently, a significant evidence gap exists regarding the impact of lifestyle interventions on this marker. Rigorous randomized controlled trials (RCTs) directly comparing the effects of balanced dietary, integrated exercise, and their combination on both 1,5-AG and CVD risk factors are lacking. It remains unclear whether a multimodal intervention confers synergistic advantages for improving short-term glycemic variability and the overall CVD risk profile.

Therefore, this study uses a combination of cross-sectional untargeted serum metabolomics and a RCT to clarify the clinical application value of serum 1,5-AG in populations with normal glucose tolerance, PDM, and DM, as well as to compare the efficacy of isolated and combined balanced dietary and integrated exercise interventions in elevating serum 1,5-AG levels and improving CVD risk factors in PDM patients, an objective that carries significant public health implications.

## 2. Materials and Methods

### 2.1. Study Design

**Figure 1:**
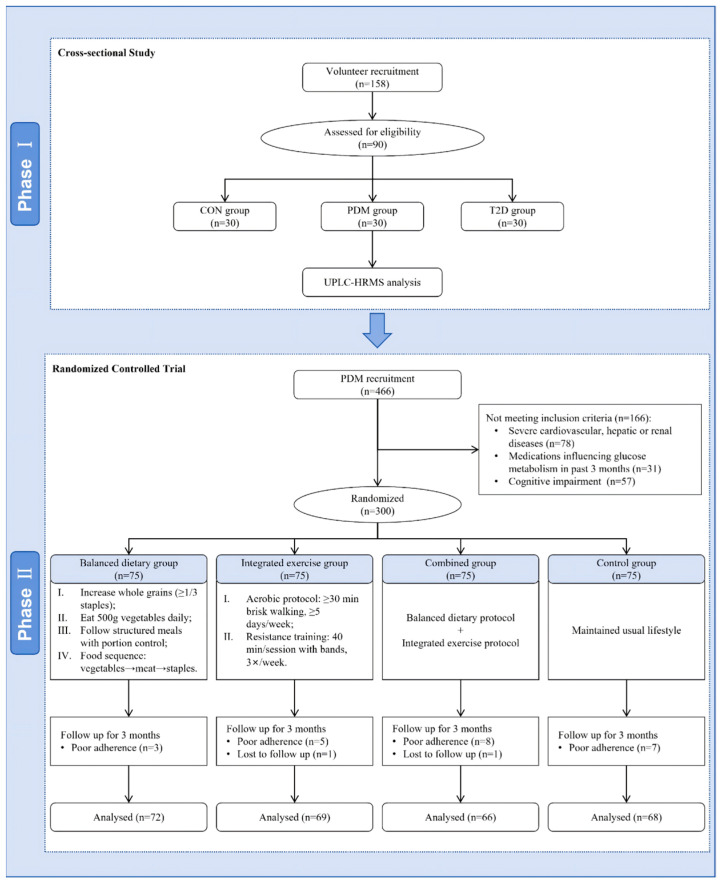
Flow diagram of the study design.

This study adopted a two-stage translational design (Figure 1). In the first stage, an exploratory analytical strategy was employed in the cross-sectional metabolomics analysis, aiming to unbiasedly screen for metabolites associated with diabetes progression. The second stage consisted of an RCT, whose objective was not to validate all the findings, but to select the single biomarker with the highest translational potential based on the first-stage results, and verify its responsiveness to lifestyle interventions. Ethical approval was obtained, the study adhered to the Declaration of Helsinki, and reporting followed the CONSORT 2025 guidelines (Table S1). All participants provided written informed consent. The study was approved by the Ethics Committee of Lianshui People’s Hospital Affiliated to Kangda College of Nanjing Medical University (Approval No. 20220401-2) on 1 April 2022.

#### 2.1.1. Cross-Sectional Study

In September 2022, 30 individuals with normoglycemia (CON), 30 individuals with PDM, and 30 individuals with DM were enrolled from Lianshui County, Jiangsu, China.

- Sample size

During untargeted metabolomics discovery, we limited multivariate models to exploratory pattern recognition and candidate biomarker screening instead of individual metabolite hypothesis testing. Given no unified sample size formula for high-dimensional multi-omics research, we followed recommendations from systematic reviews and methodological literature on multivariate prediction model design and biomarker discovery, which specify 10 samples per group (n = 10) as the minimum to boost univariate test power when intergroup metabolic differences exist [16,18,19,20]. We used univariate power calculations as the lower bound, referenced the 20–40 samples per group empirical range from prior similar studies, and adopted rigorous cross-validation, false discovery rate (FDR) control and external validation to mitigate false-positive and overfitting risks with limited sample sizes. To mitigate the confounding effects of sex and age, participants were stratified into three age groups (40–49, 50–59, and ≥60 years) with equal sample sizes (1:1:1 ratio), and a 1:1 male-to-female ratio was maintained within each subgroup.

- Inclusion and exclusion criteria

Diagnostic criteria adhered to the 2022 American Diabetes Association (ADA) standards: PDM was defined as fasting blood glucose (FBG) 6.1–6.9 mmol/L and/or glycated hemoglobin (HbA1c) 5.7–6.4% (39–46 mmol/mol); DM as FBG ≥ 7.0 mmol/L and/or HbA1c ≥ 6.5% (48 mmol/mol); and normoglycemic as FBG < 6.1 mmol/L and HbA1c < 5.7% (39 mmol/mol). Exclusion criteria included: (i) severe CVD, hepatic, or renal comorbidities; (ii) psychiatric disorders; (iii) pregnancy or lactation; (iv) use of medications affecting glucose metabolism within the preceding three months.

#### 2.1.2. RCT

The second-phase study was a 3-month, open-label, parallel-group, randomized controlled intervention trial conducted from September 2022 to March 2023. Participants were enrolled from Lianshui County, Jiangsu, China, and randomized at a 1:1:1:1 ratio to four groups (balanced dietary group, D group; integrated exercise group, E group; balanced dietary + integrated exercise group, D + E group; and control group, C group), via a computer-generated sequence. The sequence was produced by an independent statistician uninvolved in recruitment or intervention delivery, was stratified by age group (40–49, 50–59, ≥60 years) and sex, with permuted block randomization (block size = 8) within each stratum; allocation concealment was ensured using sequentially numbered, opaque, sealed envelopes prepared by the statistician and held by the site research coordinator. Outcome assessors performing laboratory analyses were blinded to group allocation. All participants received PDM management health education before the trial.

- Sample size

The sample size was calculated with reference to Ruan et al., and using formula for two independent groups in a two-tailed test: N = 2(Z_1−α/2_ + Z_β_)^2^σ^2^/d^2^, where Z_α/2_ = 1.96 (α = 0.05), power = 80%, σ = 0.67 [standard deviation (SD) of FBG], and d = 0.34 [21]. After adjusting for a 20% anticipated attrition rate, the final required sample size was 75 participants per group. L.G. generated the random allocation sequence and the method.

- Inclusion and exclusion criteria

Inclusion criteria were: (i) ≥40 years and diagnosed with PDM; (ii) intact cognitive function and verbal communication capacity; (iii) voluntary participation with informed consent. Exclusion criteria were: (i) confirmed DM diagnosis; (ii) physical mobility impairments; (iii) severe CVD, hepatic, or renal comorbidities; (iv) pregnancy or lactation; (v) recent use of medications known to influence glucose metabolism within the preceding three months, such as metformin and glucocorticoids.

- Intervention

The C group maintained their usual lifestyle and received baseline PDM education.

The balanced dietary recommendation followed the Dietary Guidelines for Chinese Residents (2022), with individualized daily energy calculated by gender, age, body mass index (BMI), and physical activity levels. Key advice included increasing whole grains to ≥1/3 of staple foods, consuming 500 g fresh vegetables daily, implementing structured portion-controlled meals, and adopting vegetable-first-meat-second-staples-last eating sequences.

Participants assigned to integrated exercise regimens received pedometers and resistance bands, performing brisk walking (≥30 min/session, ≥5 days/week) and resistance training (40 min/session, 3 times/week).

To enhance participant adherence, a WeChat group was established for each intervention arm for real-time communication, with the monitoring protocol applied uniformly and consistently across all three intervention groups to ensure equitable comparisons. Healthcare staff provided daily reminders for activity and dietary photo submissions via WeChat check-ins and conducted biweekly in-person follow-up visits to monitor compliance and address practical challenges. This combined strategy ensured real-time oversight and participant engagement, but lacked continuous quantitative metrics to support formal dose–response analysis.

### 2.2. Sample Collection

#### 2.2.1. Basic Information Survey

At enrollment, trained staff conducted baseline assessments, including anthropometrics, laboratory tests, and dietary evaluations via a validated food frequency questionnaire (FFQ) administered pre-/post-intervention to quantify 3-month retrospective dietary intake. Biweekly dietitian-led in-person counseling and daily dietary photo submissions via WeChat minimized recall bias effectively.

#### 2.2.2. Venous Blood Collection and Storage

Venous blood (2 mL) was collected by trained medical personnel after an 8–12 h overnight fast. In the first phase, untargeted metabolomics measurements including 1,5-AG were performed on these samples; in the second phase, 1,5-AG was quantified independently using the same assay method as specified below. FBG levels were measured using the hexokinase method, HbA1c was quantified via the ultra-high-performance liquid chromatography, 1,5-AG was quantified using an enzyme-linked immunosorbent assay (ELISA) (Text S1), and serum 1,5-AG concentrations are reported in ng/L as per the calibration of the specific ELISA kit used, with caution advised when comparing these absolute values with those obtained via different methodological platforms such as enzymatic assays since the absolute concentrations are method-dependent.

### 2.3. UHPLC-HRMS Analysis

A comprehensive description of the materials and the coupled with high-resolution mass spectrometry (UHPLC-HRMS) analytical protocol is outlined in the Texts S2 and S3. The UHPLC–HRMS analysis was carried out using a Vanquish UHPLC™ system (Thermo Fisher Scientific, Waltham, MA, USA) hyphenated to an Orbitrap Exploris 120 mass spectrometer (Thermo Fisher Scientific, Waltham, MA, USA). Chromatographic separation was conducted on a Vanquish™ UHPLC system. Gradient elution was carried out on a BEH Amide column (2.1 mm × 50 mm, 1.7 μm particle size) from Waters ACQUITY UHPLC (Milford, MA, USA) installed on the Thermo Fisher system. The mobile phase consisted of phase A (water containing 25 mmol/L ammonium acetate and 25 mmol/L ammonia water) and phase B (acetonitrile). The autosampler temperature was maintained at 4 °C with an injection volume of 2 μL. Mass spectrometric data acquisition in both full-scan and MS/MS modes was conducted using an Orbitrap Exploris 120 mass spectrometer controlled by Xcalibur software (V4.4, Thermo Fisher Scientific, Waltham, MA, USA). Electrospray ionization (ESI) was operated in positive and negative ion modes with spray voltages set to 3.8 kV (positive) and −3.4 kV (negative), respectively. The sheath gas and auxiliary gas flow rates were 50 Arb and 15 Arb, respectively. The ion transfer tube temperature was 320 °C. Full-scan spectra were acquired at a resolution of 60,000, while MS/MS spectra were collected at a resolution of 15,000 with stepped normalized collision energies (SNCE) of 20%, 30%, and 40%.

### 2.4. Outcomes

The primary endpoints of this randomized trial were changes in established clinical glycemic parameters, specifically FBG. In addition, based on the discovery phase, the change in serum biomarker level was pre-specified as a key exploratory endpoint to investigate the pathway-specific response to lifestyle intervention.

Secondary outcomes included several anthropometric and hemodynamic measures associated with cardiometabolic risk: body weight, BMI, waist circumference (WC), blood pressure, and the cumulative incidence of DM. It is noted that this study did not assess other components of a comprehensive cardiovascular risk profile, such as blood lipids or inflammatory markers.

### 2.5. Quality Control

Untargeted metabolomics used pooled quality control (QC) samples (equal-volume supernatant mix) analyzed alongside 10 replicates. Principal component analysis (PCA), an unsupervised pattern recognition method, was first applied to visualize the intrinsic clustering of samples. Subsequently, supervised orthogonal partial least squares-discriminant analysis (OPLS-DA) was conducted to maximize the visualization of between-group differences and ultimately identify discriminatory biomarkers.

All staff completed standardized training and competency testing before the study, strictly following predefined inclusion/exclusion criteria to reduce selection bias. Data were entered using EpiData V3.1 with double-entry verification, and any inconsistencies or missing data were resolved by contacting investigators to ensure completeness, with failed QC questionnaires being systematically excluded. Given the study’s pragmatic design and short duration, confirmatory testing was not performed.

### 2.6. Statistical Analysis

Raw data from untargeted metabolomics were converted to mzXML format using ProteoWizard software, V3.0.18332 followed by peak detection, extraction, and alignment via R V4.4.1 software. Metabolite annotation was performed using the BiotreeDB V3.0 database. To mitigate potential overfitting in the OPLS-DA model, permutation tests (n = 200) were performed by randomly shuffling class labels to generate R^2^ (goodness-of-fit) and Q^2^ (predictive ability) values for validation. Overfitting was excluded if either criterion was met: (i) all permuted Q^2^ values were lower than the original Q^2^; or (ii) the Q^2^ regression line intercept was <0. To control for false discovery, differential metabolites were screened based on a variable importance in the projection (VIP) score > 1 from the OPLS-DA model and an FDR-adjusted *p*-value (*q*-value) < 0.05, with *p*-values derived from Student’s *t*-tests and adjusted using the Benjamini–Hochberg procedure [22,23]. Receiver operating characteristic (ROC) curves were plotted to evaluate diagnostic accuracy using the area under the curve (AUC), with metabolic pathway analysis conducted via the Kyoto Encyclopedia of Genes and Genomes (KEGG) database. To correct for potential overfitting and obtain a robust estimate of the biomarker’s performance, the AUC was calculated with 10-fold cross-validation repeated twice. Both the apparent AUC (from the original model) and the validated AUC (from the internal validation) are reported. Differential metabolites were stratified by diagnostic utility: low (apparent AUC 0.5–0.7), moderate (AUC 0.7–0.9), and high (AUC ≥ 0.9) predictive power [22,23,24,25,26].

Clinical trial data were analyzed using SPSS V27.0 and Graphpad Prism V10.1.2. Primary analysis used a modified intention-to-treat (mITT) population: all randomized participants with at least one post-baseline assessment. For end-of-intervention outcomes, including serum 1,5-AG and no follow-up data from withdrawals, the analysis was effectively completed or performed per-protocol. This approach is stated explicitly for transparency, as it gives a conservative efficacy estimate in intervention adherents. Continuous variables are presented as mean ± SD for normally distributed data. Normality was assessed using the Shapiro–Wilk test. For measures meeting homogeneity of variance, paired *t*-tests or one-way ANOVA were used for group comparisons, with Dunnett’s post hoc tests for multiple comparisons. Variables violating variance homogeneity were analyzed via Welch’s ANOVA with Dunnett’s T3 post hoc adjustments. Categorical data are expressed as frequencies (counts), analyzed by Chi-square tests or Fisher’s exact test. All statistical tests were two-tailed, with a significance level of α = 0.05. Data from participants who withdrew from the intervention were excluded. All analyses were conducted based on available data from those who completed the study.

## 3. Results

### 3.1. Cross-Sectional Study Results

#### 3.1.1. Participant Characteristics

A total of 90 participants were recruited, and their characteristics are summarized in Table 1. Significant differences (*p* < 0.05) were observed across the groups in FBG, HbA1c, systolic and diastolic blood pressure, body weight, WC, BMI, and family history of diabetes. In contrast, no significant differences (*p* > 0.05) were found in age, height, sex, or educational attainment.

#### 3.1.2. Untargeted Metabolic Analysis

Stable internal standard (IS) retention time and response intensity were confirmed by low variation in QC peak heights (Figure S1), and all QCs clustered within ±2 SD of the central line (Figure S2), demonstrating robust data reliability.

PCA showed serum sample overlap (Figure S3). OPLS-DA filtered classification-independent variables (Figure S4). Permutation tests ruled out overfitting (Figure S5), confirming distinct metabolite profiles among groups. Number of differential metabolites in Table S2. These metabolites were annotated and classified using the Human Metabolome Database (HMDB), revealing predominant enrichment in carbohydrates, amino acids and their metabolites, fatty acids and derivatives, organic acids and derivatives, and alkaloids. A Venn diagram illustrating shared and unique differential metabolites across groups is presented in Figure S6.

#### 3.1.3. ROC Analysis

ROC analysis was performed on the 53 differential metabolites identified via Venn diagram analysis, yielding a panel of biomarkers with AUC values > 0.5 for distinguishing the three groups. Using the criteria of AUC > 0.7 and retrievability in both the HMDB and KEGG COMPOUND databases, the AUCs of potential biomarkers were calculated to assess their diagnostic performance (Table S3). After Benjamini–Hochberg FDR correction, only two metabolites, taurine and sulfasalazine, met these stringent criteria in the CON vs. PDM comparison (Table S3). However, after internal validation, taurine’s corrected AUC was 0.56 (95% CI: 0.27–0.86) for PDM vs. DM, which falls below our pre-specified threshold of 0.7 for a robust biomarker. For sulfasalazine, the *q*-values were 0.007 (apparent AUC = 0.80, 95% CI: 0.69–0.92) for CON vs. PDM, <0.001 (apparent AUC = 0.88, 95% CI: 0.80–0.97) for PDM vs. DM, and <0.001 (apparent AUC = 0.98, 95% CI: 0.95–1.00) for DM vs. CON. Following cross-validation, the corrected AUCs for these comparisons remained above 0.7. These results suggest that sulfasalazine may serve as a robust metabolic signature for the very early stage of prediabetes.

**Figure 2:**
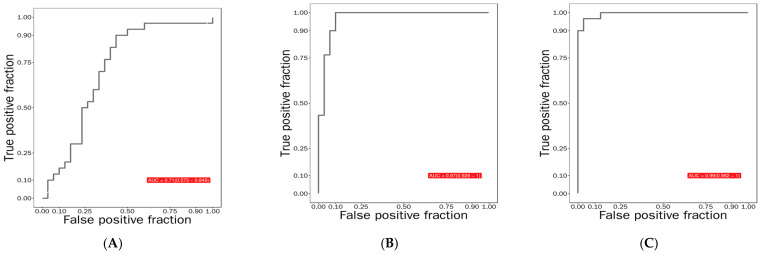
ROC curves of 1,5-AG among groups. (**A**) apparent AUC of CON vs. PDM; (**B**) apparent AUC of PDM vs. DM; (**C**) apparent AUC of CON vs. DM. Note: ROC, receiver operating characteristic; PDM, prediabetes; DM, diabetes mellitus; CON, control group; 1,5-AG, 1,5-anhydroglucitol.

Under the slightly looser yet commonly used exploratory screening criteria (VIP > 1.0 and unadjusted *p* < 0.05), a total of 8 differential metabolites were identified (Table S3), providing a more comprehensive insight into the metabolic reprogramming underlying disease progression. Notably, 1,5-AG exhibited overwhelmingly significant diagnostic efficacy in distinguishing PDM from DM (*q* < 0.001, apparent AUC = 0.97, 95% CI: 0.93–1.00; corrected AUC = 0.94, 95% CI: 0.83–1.00), demonstrating exceptional and robust performance (Figure 2). While internal validation confirmed several metabolites with high discriminatory power, such as phellopterin, 1,5-AG was selected as the prime candidate for longitudinal validation owing to its top-tier validated AUC, well-established physiological link to glycemia, and widespread clinical application for short-term glycemic excursion monitoring, all of which greatly enhance its immediate translational potential. Based on these findings, 1,5-AG was selected for longitudinal validation in an RCT.

Using criteria of impact > 0.1 and −ln(*p*) > 1, key metabolic pathways with high relevance were identified (Figure S7). The analysis revealed that between the CON and PDM groups, the significantly enriched pathways included alanine, aspartate and glutamate metabolism; taurine and hypotaurine metabolism; aminoacyl-tRNA biosynthesis; tyrosine metabolism; and D-glutamine and D-glutamate metabolism. Between the PDM and DM groups, the enriched pathway was the citrate cycle (tricarboxylic acid cycle, TCA cycle) and taurine and hypotaurine metabolism. Between the CON and DM groups, the enriched pathways comprised glycine, serine and threonine metabolism; taurine and hypotaurine metabolism; alanine, aspartate and glutamate metabolism; linoleic acid metabolism; and the pentose phosphate pathway.

### 3.2. RCT Results

#### 3.2.1. Baseline Characteristics

Of the 466 individuals with PDM initially assessed, 166 (35.6%) were excluded prior to randomization, primarily due to the presence of severe comorbidities, use of medications affecting glucose metabolism, or cognitive impairment. This exclusion rate reflects the application of stringent safety and protocol adherence criteria necessary for a lifestyle intervention trial, and it indicates that the enrolled participants represent a subset of the broader PDM population without these complicating factors.

Baseline characteristics (Table 2) and dietary intake (Table S4) were comparable across groups (all *p* > 0.05). During the trial, 275 participants completed the study, with dropout rates were 4.0% (D), 8.0% (E), 12.0% (D + E), and 9.3% (C), with no significant difference across groups (χ^2^ = 3.000, *p* = 0.392). Attrition rates were slightly higher in the D + E (12.0%) compared to E (8.0%) and C group (C, 9.3%). Withdrawals were primarily due to loss to follow-up or poor adherence unrelated to adverse events, this may reflect the greater time and effort demands associated with active intervention protocols. No adverse events were observed.

Although powered for conventional glycemic endpoints, this trial underwent post hoc power analysis to evaluate sensitivity for detecting the observed effect on the exploratory biomarker 1,5-AG. Using the 3-month mean difference (80 ng/L) and pooled SD (35.4 ng/L) between the combined intervention group (D + E, n = 66) and control group (C, n = 68), the calculated Cohen’s d was 1.377. With α = 0.05 (two-sided) and the achieved sample size, post hoc power exceeded 99.9% (G*Power V3.1.9.7), confirming high sensitivity for testing the hypothesis that 1,5-AG levels respond to lifestyle intervention.

#### 3.2.2. Primary Outcomes

Post-intervention changes in FBG across the four groups are summarized in Table 3. FBG levels in the C group increased relative to baseline over the 3-month period, with a change of 1.4 ± 1.8 mmol/L and *p* < 0.05. Among the three lifestyle intervention groups, significant FBG reductions were observed in the D and D + E groups (D: ∆FBG = −0.3 ± 1.0 mmol/L; D + E: ∆FBG = −0.3 ± 1.0 mmol/L; both *p* < 0.05), whereas no significant FBG changes were detected in the E group (∆FBG = 0.1 ± 1.0 mmol/L; *p* = 0.639). Additionally, compared with baseline data, FBG levels in the control group increased significantly (6.7 ± 1.7 vs. 5.3 ± 0.6, mmol/L; *p* < 0.001). By contrast, both the D and D + E group exhibited a significant reduction (D: 5.1 ± 1.0 vs. 5.4 ± 0.5, mmol/L; D + E: 5.1 ± 0.9 vs. 5.4 ± 0.6, mmol/L; all *p* < 0.05). Notably, following 3 months of intervention, FBG and HbA1c levels in all three lifestyle intervention groups were significantly lower than those in the C group (*p* < 0.05).

In alignment with these clinical improvements, and as an exploratory mechanistic finding, serum levels of 1,5-AG at 3 months were significantly different among the four groups (*p* < 0.05, Table 3 and Figure S8). The highest level was observed in the D + E group (217.7 ± 58.2 ng/L), followed by the D group (183.2 ± 60.7 ng/L), while the E (156.5 ± 51.3 ng/L) and C (138.4 ± 57.0 ng/L) groups showed the lowest values. Post hoc pairwise comparisons revealed that all inter-group differences were statistically significant, except for that between the E and C groups. This pattern indicates that the dietary component was the primary driver of increased serum 1,5-AG, while the combined intervention yielded the greatest effect.

#### 3.2.3. Changes in CVD Risk Factors

Table 3 showed that among the three lifestyle intervention groups, all three lifestyle intervention groups exhibited significant decreases in both FBG and HbA1c (all *p* < 0.05). In contrast, the C group demonstrated increases in both FBG and HbA1c [∆FBG = 1.4 ± 1.8, mmol/L; ∆HbA1c = 0.3 ± 0.9% (19.9 ± 9.4, mmol/mol); *p* < 0.05]. Additionally, compared with baseline data, HbA1c levels in the control group increased significantly [6.3 ± 0.9 vs. 6.0 ± 0.3% (45.2 ± 9.3 vs. 41.5 ± 3.2 mmol/mol); *p* = 0.002]. By contrast, only the D + E group exhibited a significant reduction in HbA1c relative to baseline [5.7 ± 0.5 vs. 5.9 ± 0.2% (38.9 ± 5.3 vs. 40.7 ± 2.1 mmol/mol); *p* = 0.005], while the other two intervention groups showed a non-significant downward trend (*p* > 0.05).

Weight, WC, and BMI significantly decreased in D and D + E groups vs. baseline (*p* < 0.05) (Table 3), but not in E or C groups. Only C group showed increased SBP (*p* < 0.05). No significant changes were detected in SBP or DBP among the other groups. While after 3 months, there were no statistically significant differences between groups in changes in body weight, WC, BMI and blood pressure.

#### 3.2.4. Relationship of 1,5-AG with CVD Risk Factors

**Figure 3:**
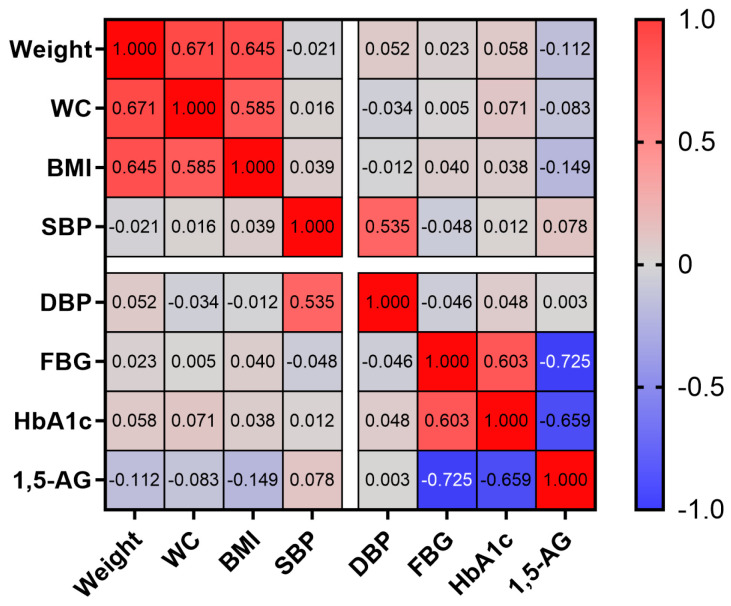
Heatmap of the Correlation Between 1,5-AG and CVD Risk Factors. Note: 1,5-AG, 1,5-anhydroglucitol; WC, waist circumference; BMI, body mass index; SBP, systolic blood pressure; DBP, diastolic blood pressure; FBG, fasting blood glucose; HbA1c, hemoglobin A1c.

Pearson correlation analysis revealed distinct associations between serum 1,5-AG levels and various CVD risk factors (Figure 3). Specifically, a strong inverse correlation was observed between 1,5-AG and FBG (r = −0.725, *p* < 0.001), along with a significant negative correlation with HbA1c (r = −0.659, *p* < 0.001), indicating a close relationship between glycemic control and circulating 1,5-AG concentrations. In contrast, BMI showed only a weak inverse association with 1,5-AG (r = −0.149, *p* = 0.014). No statistically significant correlations were detected between 1,5-AG and body weight, WC, SBP, or DBP (all *p* > 0.05). While observed 1,5-AG changes correlated with improved glycemia, its direct association with broader cardiovascular risk factors, such as blood pressure, was not evident in our study. These findings suggest 1,5-AG is a specific biomarker for glycemic flux rather than a general marker of integrated cardiovascular risk, highlighting the need for future studies with comprehensive lipid and inflammatory marker panels to clarify potential cardiometabolic links.

#### 3.2.5. Incidence of DM

Pearson’s chi-square test showed a significant overall difference in DM incidence across the four groups (D: 9.7%, E: 8.7%, D + E: 7.6%, C: 22.1%; χ^2^ = 8.806, *p* = 0.032; Table S5), though pairwise comparisons with Bonferroni correction yielded no statistically significant differences between any two groups (all *p* > 0.05). Numerically, the D + E group had the lowest DM incidence (7.58%), representing a 65.6% relative risk reduction versus the control group (22.06%, *p*′ = 0.209); this lack of significance may stem from limited statistical power due to strict multiplicity adjustment. The control group’s 3-month incidence is consistent with reported rates in intensively monitored high-risk prediabetes cohorts, reflecting the aggressive natural history of untreated dysglycemia in this population [27].

#### 3.2.6. Dietary Intake Changes

Table S6 shows dietary intake changes. Analysis of dietary intake at the 3-month endpoint revealed that total energy intake was significantly lower in the D (baseline: 801.6 ± 521.2 kcal; post-intervention: 1517.8 ± 410.3 kcal; Δ: −283.7 ± 534.5 kcal) and D + E (baseline: 1797.4 ± 436.3 kcal; post-intervention: 1572.0 ± 375.5 kcal; Δ: −225.4 ± 527.7 kcal) groups compared to the E (baseline: 1820.3 ± 523.5 kcal; post-intervention: 1813.7 ± 386.7 kcal; Δ: −6.6 ± 572.5 kcal) and C (baseline: 1933.5 ± 526.0 kcal; post-intervention: 1866.4 ± 364.4 kcal; Δ: −67.1 ± 584.2 kcal) groups (*p* < 0.05; Table S6). When comparing the changes in energy intake from baseline to 3 months, significant reductions were observed in the D group compared to both the E and C groups, and in the D + E group compared to the E group (*p* < 0.05; Table S6).

Carbohydrate intake decreased in D (Δ: −59.8 ± 86.5 g) and D + E (Δ: −57.0 ± 69.2 g) groups compared to that in the E (Δ: −7.2 ± 87.4 g) and C (Δ: −16.6 ± 90.2 g) groups (*p* < 0.001; Table S6), while fat and protein showed no within-group changes (*p* > 0.05). Notably, the pattern of change indicates that the observed reduction in total energy intake was driven almost entirely by the decrease in carbohydrate consumption, rather than by changes in fat or protein. Refined staples, tubers, and pulses mirrored carbohydrate patterns. Vegetable intake rose uniquely in D (Δ: 159.4 ± 242.7 g) group (*p* < 0.001), surpassing E (Δ: 0.1 ± 198.0 g) and C increases (Δ: 22.1 ± 164.1 g). Other food categories showed no significant changes across groups.

## 4. Discussion

Our two-phase design enabled us to first identify 1,5-AG as a sensitive biomarker for dysglycemic states, and then to validate it in a clinical intervention. We demonstrated that a combined lifestyle intervention was uniquely effective, not only significantly elevating serum 1,5-AG levels but also improving multiple CVD risk factors in individuals with PDM, with superior outcomes compared to either intervention alone. These findings establish the value of 1,5-AG for monitoring short-term glycemic fluctuations and underscore the synergistic cardiometabolic benefits of multimodal lifestyle modification in this high-risk population.

When comparing the metabolic profiles among individuals with normal glucose tolerance, PDM, and DM, certain metabolites exhibited significant alterations along the disease continuum [28,29,30]. In a related study, Al-Sulaiti et al. investigated metabolic changes during the progression from obesity to diabetes and identified disruptions in several pathways, including glycolysis, gluconeogenesis, and pyruvate metabolism (involving 1,5-AG), histidine metabolism, and phospholipid metabolism [28]. Consistent with these findings, our study identified nine altered metabolites associated with glucose metabolism. Among these, 1,5-AG, D-mannose, D-fructose, D-tagatose, and D-glucose began to change as early as the PDM stage. However, histidine and phospholipid pathways were not significantly enriched in our PDM cohort (Figure S7), but related metabolites in the broader differential pool suggest possible involvement. Differences in pathway enrichment across studies may reflect variation in population characteristics, disease stage, or analytical focus.

In the exploratory phase, strict FDR correction demonstrated the importance of sulfasalazine as a potential early biomarker for prediabetes. From the perspective of translational medicine and clinical utility, we decided to focus validation resources on 1,5-AG [31,32]. This decision was based on multiple considerations. First, its diagnostic performance as a biomarker for disease progression, with an AUC > 0.90 for PDM vs. DM, confers strong clinical differentiation value for identifying high-risk individuals. Second, its glucose-regulated physiological mechanism provides robust biological plausibility for dynamic intervention efficacy monitoring, a hypothesis validated in the present RCT. 1,5-AG, as a predominant polyol in human physiology, its plasma levels reflect renal handling: filtered via glomeruli and reabsorbed in renal tubules. Due to its limited metabolism in the body, its concentration remains stable. However, when blood glucose exceeds the renal glucose threshold, 1,5-AG reabsorption in the kidneys is competitively inhibited by glucose, leading to a decline in serum levels [31]. These results underscore that dysregulation of glucose metabolism is a pivotal mechanism driving the transition from normoglycemia to diabetic hyperglycemia. Given that 1,5-AG is recognized as a marker of short-term glycemic control and glucose fluctuations, the PDM stage represents a critical window for preventing the onset of diabetes and its associated complications [29].

This study further confirms a significant inverse correlation between serum 1,5-AG and key CVD risk factors. A previous real-world cohort study reported that each one-standard deviation (5.5 μg/mL) decrease in 1,5-AG was associated with a 14% increased risk of all-cause mortality (95% CI: 1.03–1.26) and a 25% increased risk of CVD mortality (95% CI: 1.03–1.52) [33]. In line with this, our findings demonstrate strong negative correlations of serum 1,5-AG with FBG and HbA1c, a weak negative correlation with BMI, and no significant association with other CVD risk factors such as blood pressure. However, whether the observed association between 1,5-AG and glycemic markers is fully independent of HbA1c-reflected average glycemic levels remains debated, as our correlation analyses cannot confirm its predictive value beyond conventional metrics; future studies should employ multivariable regression models in larger longitudinal cohorts to definitively explore this question.

The synergistic benefits of combined balanced dietary and integrated exercise in DM are well-established and are reflected in the ADA’s Standards of Care in Diabetes—2025 [34]. However, robust clinical evidence supporting this synergy in individuals with PDM remains limited. A two-year real-world study by Viswanathan et al. demonstrated that an integrated lifestyle intervention significantly reduced diabetes incidence compared to standard care (9.1% vs. 34.0%, *p* = 0.004) in a PDM population [27]. Extending these findings, our study isolated the effects of a balanced diet, integrated exercise, and their combination. We observed that the combined intervention yielded the most substantial improvements in serum 1,5-AG levels and CVD risk factors. This finding underscores the critical importance of multimodal lifestyle modification in PDM management.

Our balanced, portion-controlled dietary intervention aligned with national recommendations and still produced marked shifts in circulating metabolites and glycemic control when combined with exercise, highlighting that even moderate dietary modification can reprogram metabolic networks. Other patterns, such as ketogenic diets, which differ fundamentally in macronutrient composition and physiological targets yet exert powerful effects on systemic metabolism, inflammation and neuroimmune function, further illustrate that distinct dietary approaches can converge on improved metabolic health via partially overlapping pathways [35]. A key dietary analysis finding is the specific macronutrient shift from this balanced intervention: total energy intake fell in the diet (D) and diet-exercise (D + E) groups, with post hoc analysis attributing this reduction solely to decreased carbohydrate intake rather than fat or protein consumption (Table S6). This pattern indicates that the dietary protocol succeeded via targeted carbohydrate moderation (whole grain promotion, portion control, meal sequencing) rather than calorie restriction, a critical distinction, as this approach may confer metabolic benefits for prediabetes glycemic control. The unique increase in vegetable intake in Group D further highlights the improved dietary quality, complementing reduced refined carbohydrate intake. Within this context, we observed that serum 1,5-AG responded robustly to the multimodal lifestyle intervention and correlated closely with established glycemic markers over three months, during which the control group showed rapid diabetes progression with an incidence of 22.06%, whereas the intervention yielded a 65.6% relative reduction in diabetes onset despite limited power for multiplicity-adjusted significance. These findings suggest a potential benefit and warrant confirmation in a larger, longer-term trial.

Several limitations of this study should be acknowledged, which collectively affect the interpretation and generalizability of our findings. First, the generalizability of our results is limited by participant selection; the 35.6% enrollment exclusion rate due to stringent safety and eligibility criteria, including severe comorbidities or relevant medications, and though necessary to ensure safety and isolate lifestyle intervention effects, this limits direct applicability to all PDM individuals, especially those with multimorbidity or complex pharmacotherapy. Second, the study has inherent methodological constraints, including a relatively modest sample size, limited follow-up duration, and the absence of baseline 1,5-AG measurements in the RCT, owing to pandemic-related logistical constraints. These limitations preclude an assessment of the long-term sustainability of lifestyle improvements and a comprehensive evaluation of their effects on existing CVD risk factors. Additionally, the AUC values reported in the cross-sectional discovery phase, while presented with confidence intervals, were derived from the same dataset used for feature selection without internal validation, which may lead to an optimistic estimate of their diagnostic performance. Fourth, the analysis of endpoint 1,5-AG was based on completers; while this provides a valid estimate of efficacy in adherent participants, it may overestimate the effect size if applied to the entire randomized population. An additional consideration is the geographic and ethnic specificity of our cohort, drawn from a Chinese population. While this provides valuable insights within this context, the generalizability of the metabolic signatures we identified to other ethnic groups requires further investigation. Future studies should therefore seek to validate these findings in larger, more heterogeneous cohorts over extended periods in real-world settings to enhance their generalizability and clinical applicability.

## 5. Conclusions

Taken together, the coherence and magnitude of changes across fasting glucose, HbA1c, 1,5-AG and key metabolomic signatures support the biological plausibility and translational potential of multimodal lifestyle modification as a core strategy for diabetes prevention and nominate serum 1,5-AG as a promising auxiliary biomarker for short-term glycemic monitoring and future comparative studies of different dietary patterns on specific metabolic pathways such as taurine–hypotaurine metabolism. Future research should aim to validate these findings in longer-term trials and explore the molecular mechanisms underlying the observed synergy.

## Figures and Tables

**Table 1:** The characteristics of cross-sectional study participants.

Variable	CON (n = 30)	PDM (n = 30)	DM (n = 30)	F	*p*
Age, years	54.3 ± 9.0	55.8 ± 7.7	55.9 ± 8.4	0.343	0.711
Educational level				-	0.999
Illiterate, n (%)	6 (20.0)	6 (20.0)	6 (20.0)		
Primary school, n (%)	10 (33.3)	10 (33.3)	11 (36.7)		
Junior high school, n (%)	11 (36.7)	10 (33.3)	9 (30.0)		
Senior high school, n (%)	3 (10.0)	4 (13.3)	4 (13.3)		
Family history of DM				-	<0.001
No, n (%)	29 (96.7)	24 (80.0)	17 (56.7)		
Yes, n (%)	1 (3.3)	6 (20.0)	13 (43.3)		
Height, cm	159.6 ± 7.0	160.4 ± 8.3	159.9 ± 9.6	0.060	0.941
Weight, kg	60.4 ± 8.9	69.9 ± 13.2	68.7 ± 13.0	5.683	0.005
WC, cm	80.6 ± 7.1	89.9 ± 10.0	88.1 ± 12.0	7.344	0.001
BMI, kg/m^2^	23.7 ± 2.6	27.0 ± 3.6	26.7 ± 3.8	9.102	<0.001
SBP, mmHg	126.6 ± 19.4	136.6 ± 13.7	137.8 ± 18.9	3.670	0.030
DBP, mmHg	78.8 ± 14.3	84.5 ± 8.7	85.9 ± 9.8	3.224	0.045
FBG, mmol/L	4.4 ± 0.2	6.0 ± 0.6	11.2 ± 2.7	147.083	<0.0001
HbA1c, %	4.7 ± 0.1	6.1 ± 0.2	10.1 ± 1.9	195.504	<0.0001
HbA1c, mmol/mol	27.8 ± 1.2	42.8 ± 2.1	86.8 ± 20.7	195.504	<0.0001

Continuous variables: mean ± SD (normal); categorical variables: n (%). Note: CON, normoglycemic control group; PDM, prediabetes; DM, diabetes mellitus; WC, waist circumference; BMI, body mass index; SBP, systolic blood pressure; DBP, diastolic blood pressure; FBG, fasting blood glucose; HbA1c, hemoglobin A1c.

**Table 2:** Baseline RCT participant characteristics.

Variable	D (n = 72)	E (n = 69)	D + E (n = 66)	C (n = 68)	F/χ^2^	*p*
Sex					2.272	0.518
Male, n (%)	30 (41.7)	28 (40.6)	27 (40.9)	35 (51.5)		
Female, n (%)	42 (58.3)	41 (59.4)	39 (59.1)	33 (48.5)		
Age, years	59.8 ± 6.6	59.7 ± 5.4	59.9 ± 6.4	59.3 ± 6.1	0.098	0.961
Educational level					13.953	0.247
Illiterate, n (%)	17 (23.6)	17 (24.6)	22 (33.3)	15 (22.1)		
Primary school, n (%)	24 (33.3)	12 (17.4)	12 (18.2)	21 (30.9)		
Junior high school, n (%)	24 (33.3)	29 (42.0)	27 (40.9)	22 (32.4)		
Senior high school, n (%)	6 (8.1)	10 (14.5)	4 (6.1)	10 (14.7)		
College and above, n (%)	1 (1.4)	1 (1.4)	1 (1.5)	0 (0.0)		
Family history of DM					4.710	0.194
No, n (%)	61 (84.7)	59 (85.5)	63 (95.5)	59 (86.8)		
Yes, n (%)	11 (15.3)	10 (14.5)	3 (4.5)	9 (13.2)		
Height, cm	158.6 ± 6.8	158.1 ± 9.3	157.8 ± 7.6	158.1 ± 9.6	0.092	0.965
Weight, kg	66.5 ± 10.6	65.8 ± 11.6	62.6 ± 10.1	65.0 ± 10.8	1.691	0.169
WC, cm	88.7 ± 10.4	86.7 ± 10.1	85.9 ± 7.9	87.5 ± 9.8	1.034	0.378
BMI, kg/m^2^	26.4 ± 3.4	26.2 ± 3.7	25.1 ± 3.3	26.2 ± 5.1	1.551	0.202
SBP, mmHg	139.8 ± 18.4	135.1 ± 16.9	133.3 ± 16.6	135.3 ± 17.6	1.762	0.155
DBP, mmHg	84.6 ± 10.0	82.1 ± 10.5	80.5 ± 9.9	81.9 ± 10.1	1.990	0.116
FBG, mmol/L	5.4 ± 0.5	5.4 ± 0.6	5.4 ± 0.6	5.3 ± 0.6	0.316	0.814
HbA1c, %	5.9 ± 0.2	5.9 ± 0.2	5.9 ± 0.2	6.0 ± 0.3	2.294	0.078
HbA1c, mmol/mol	41.5 ± 2.6	40.7 ± 2.5	40.7 ± 2.1	41.5 ± 3.2	2.294	0.078
Dietary intake						
Energy, kJ	7537.9 ± 2180.7	7616.1 ± 2190.3	7520.3 ± 1825.5	8089.8 ± 2200.8	1.111	0.345
Energy, kcal	1801.6 ± 521.2	1820.3 ± 523.5	1797.4 ± 436.3	1933.5 ± 526.0	1.111	0.345
Carbohydrates, g	300.0 ± 93.3	304.0 ± 88.3	296.2 ± 68.0	315.5 ± 75.8	0.995	0.553
Fats, g	34.0 ± 17.8	33.4 ± 16.8	35.6 ± 20.6	38.4 ± 21.6	0.925	0.429
Proteins, g	77.3 ± 24.5	80.2 ± 30.0	78.0 ± 26.3	84.7 ± 30.0	0.700	0.395

Continuous variables: mean ± SD (normal); categorical variables: n (%). Note: D, balanced dietary group; E, integrated exercise group; D + E, balanced dietary + integrated exercise group; C, control group; DM, diabetes mellitus; WC, waist circumference; BMI, body mass index; SBP, systolic blood pressure; DBP, diastolic blood pressure; FBG, fasting blood glucose; HbA1c, hemoglobin A1c.

**Table 3:** Effects of the Intervention on Serum 1,5-AG and Other Cardiometabolic Parameters.

Variable	Baseline	3-Month	t	*p*	Change	F	*p*
Weight (kg)						1.619	0.186
D (n = 72)	66.5 ± 10.6	65.3 ± 9.0 ^a^	2.202	0.031	−1.2 ± 4.6 ^a,b^		
E (n = 69)	65.8 ± 11.6	64.8 ± 9.7 ^a^	1.819	0.073	−1.0 ± 4.4 ^a,b^		
D + E (n = 66)	62.6 ± 10.1	59.7 ± 8.0 ^b^	3.613	0.001	−2.9 ± 6.5 ^a^		
C (n = 68)	65.0 ± 10.8	65.0 ± 10.2 ^a^	−0.235	0.815	0.1 ± 1.7 ^b^		
WC (cm)						1.875	0.134
D (n = 72)	88.7 ± 10.4	87.9 ± 9.1 ^a^	2.033	0.046	−0.7 ± 3.0 ^a,b^		
E (n = 69)	86.7 ± 10.1	86.1 ± 8.1 ^a,b^	1.201	0.234	−0.6 ± 3.9 ^a,b^		
D + E (n = 66)	85.9 ± 8.0	84.4 ± 6.0 ^b^	2.532	0.014	−1.5 ± 4.8 ^a^		
C (n = 68)	87.5 ± 9.8	87.8 ± 9.0 ^a^	−1.475	0.145	0.3 ± 1.4 ^b^		
BMI (kg/m^2^)						1.850	0.139
D (n = 72)	26.4 ± 3.4	25.9 ± 2.7 ^a^	2.144	0.036	−0.5 ± 1.8 ^a,b^		
E (n = 69)	26.2 ± 3.7	25.9 ± 3.0 ^a^	1.716	0.091	−0.4 ± 1.7 ^a,b^		
D + E (n = 66)	25.1 ± 3.3	23.9 ± 2.1 ^b^	3.698	<0.001	−1.2 ± 2.6 ^a^		
C (n = 68)	26.2 ± 5.1	26.2 ± 5.0 ^a^	−0.430	0.668	0.1 ± 0.6 ^b^		
SBP (mmHg)						0.235	0.872
D (n = 72)	139.8 ± 18.4	141.7 ± 16.7	−0.728	0.469	1.9 ± 12.6		
E (n = 69)	135.1 ± 16.9	135.4 ± 15.9	−0.741	0.461	0.3 ± 3.0		
D + E (n = 66)	133.3 ± 16.6	133.8 ± 14.9	−1.423	0.159	0.5 ± 2.9		
C (n = 68)	135.3 ± 17.6	136.5 ± 15.8	−2.309	0.024	1.2 ± 4.1		
DBP (mmHg)						0.391	0.76
D (n = 72)	84.6 ± 10.0	84.7 ± 9.8	−0.608	0.545	0.1 ± 1.3		
E (n = 69)	82.1 ± 10.5	82.3 ± 10.0	−0.916	0.363	0.2 ± 1.8		
D + E (n = 66)	80.5 ± 9.9	80.8 ± 8.8	−0.956	0.343	0.3 ± 2.6		
C (n = 68)	81.9 ± 10.1	82.4 ± 8.5	−1.366	0.176	0.5 ± 2.8		
FBG (mmol/L)						32.681	<0.001
D (n = 72)	5.4 ± 0.5	5.1 ± 1.0 ^a^	2.526	0.014	−0.3 ± 1.0 ^a^		
E (n = 69)	5.4 ± 0.6	5.5 ± 1.0 ^a^	−0.471	0.639	0.1 ± 1.0 ^a^		
D + E (n = 66)	5.4 ± 0.6	5.1 ± 0.9 ^a^	2.230	0.029	−0.3 ± 1.0 ^a^		
C (n = 68)	5.3 ± 0.6	6.7 ± 1.7 ^b^	−6.351	<0.001	1.4 ± 1.8 ^b^		
HbA1c (%)						9.134	<0.001
D (n = 72)	5.9 ± 0.2	5.9 ± 0.5 ^a^	0.683	0.497	−0.0 ± 0.5 ^a^		
E (n = 69)	5.9 ± 0.2	5.9 ± 0.4 ^a^	0.178	0.860	−0.0 ± 0.4 ^a^		
D + E (n = 66)	5.9 ± 0.2	5.7 ± 0.5 ^a^	2.880	0.005	−0.2 ± 0.5 ^a^		
C (n = 68)	6.0 ± 0.3	6.3 ± 0.9 ^b^	−3.205	0.002	0.3 ± 0.9 ^b^		
HbA1c (mmol/mol)						9.134	<0.001
D (n = 72)	41.5 ± 2.6	41.1 ± 5.6 ^a^	0.683	0.497	−23.9 ± 5.0 ^a^		
E (n = 69)	40.7 ± 2.5	40.6 ± 4.5 ^a^	0.178	0.860	−23.6 ± 4.7 ^a^		
D + E (n = 66)	40.7 ± 2.1	38.9 ± 5.3 ^a^	2.880	0.005	−25.3 ± 5.0 ^a^		
C (n = 68)	41.5 ± 3.2	45.2 ± 9.3 ^b^	−3.205	0.002	−19.9 ± 9.4 ^b^		
1,5-AG (ng/L) ^†^						24.612	<0.001
D (n = 72)	-	183.2 ± 60.7 ^a^					
E (n = 69)	-	156.5 ± 51.3 ^b^					
D + E (n = 66)	-	217.7 ± 58.2 ^c^					
C (n = 68)	-	138.4 ± 57.0 ^b^					

Continuous variables: mean ± SD (normal). Different lowercase letters indicate statistically significant differences between groups (Dunnett’s test for equal variances or Dunnett’s T3 test for unequal variances, *p* < 0.05). ^†^: Serum 1,5-AG baseline data unavailable due to sample collection logistical constraints; only post-intervention values are shown, with no within-group Δ values calculable; comparisons are intergroup at 3-month endpoint. Note: D balanced dietary group; E, integrated exercise group; D + E, balanced dietary + integrated exercise group; C, control group; WC, waist circumference; BMI, body mass index; SBP, systolic blood pressure; DBP, diastolic blood pressure; FBG, fasting blood glucose; HbA1c, hemoglobin A1c.

## Data Availability

The original contributions presented in this study are included in the article/Supplementary Materials. Further inquiries can be directed to the corresponding author.

## Supplementary Materials

The following supporting information can be downloaded at https://www.mdpi.com/article/10.3390/life16020198/s1, Text S1: Measurement of 1,5-AG levels; Text S2: Instruments and Chemicals; Text S3: UHPLC-HRMS Analytical Protocol; Figure S1: Representative EICs of the internal standard in QC samples; Figure S2: One-dimensional distribution plot of QC samples along the PCA-X axis; Figure S3: PCA score plots; Figure S4: OPLS-DA score plots; Figure S5: Permutation test results of OPLS-DA models; Figure S6: Venn diagram of differentially abundant metabolites among groups; Figure S7: Bubble plots of metabolic pathway analysis; Figure S8: Comparison of serum 1,5-AG levels between groups at the 3-month endpoint; Table S1: CONSORT 2025 checklist item description [36]; Table S2: Number of differential metabolites; Table S3: Significantly differential metabolites between groups in serum samples; Table S4: Baseline daily intake of food categories among RCT participants; Table S5: DM incidence in groups after 3-month intervention; Table S6: Daily dietary intake of participants before and 3-month after lifestyle intervention.

## Abbreviations

The following abbreviations are used in this manuscript:

PDM	Prediabetes mellitus
DM	Diabetes mellitus
CVD	Cardiovascular disease
1,5-AG	1,5-anhydroglucitol
RCTs	Rigorous randomized controlled trials
CON	Individuals with normoglycemia
FDR	False discovery rate
ADA	American Diabetes Association
FBG	Fasting blood glucose
HbA1c	Hemoglobin A1c
D group	Balanced dietary group
E group	Integrated exercise group
D + E group	Balanced dietary + integrated exercise group
C group	control group
SD	Standard deviation
BMI	Body mass index
FFQ	Food frequency questionnaire
ELISA	Enzyme-linked immunosorbent assay
UHPLC-HRMS	Ultra-high-performance liquid chromatography coupled with high-resolution mass spectrometry
ESI	Electrospray ionization
SNCE	Stepped normalized collision energies
WC	Waist circumference
QC	Quality control
PCA	Principal component analysis
OPLS-DA	Orthogonal projections to latent structures-discriminant analysis
VIP	Variable importance in the projection
ROC	Receiver operating characteristic
AUC	Area under the curve
KEGG	Kyoto Encyclopedia of Genes and Genomes
mITT	Modified intention-to-treat
SBP	Systolic blood pressure
DBP	Diastolic blood pressure
IS	Internal standard
HMDB	Human Metabolome Database
TCA cycle	Tricarboxylic acid cycle
