# Supplementary material for: Effects of Balanced Dietary Patterns and/or Integrated Exercise on Serum 1,5-Anhydroglucitol and CVD Risk Factors in Individuals with Prediabetes
Source: Life (Basel). 2026 Jan 25;16(2):198. doi: 10.3390/life16020198 (PMC12941816; doi:10.3390/life16020198)
Supplement: Supplementary file 1 [file life-16-00198-s001.zip › Supplementary -V2.pdf]

Text S1. Measurement of 1,5-AG levels

Serum 1,5-anhydroglucitol (1,5-AG) levels were measured using an enzyme-linked immunosorbent assay (ELISA). A commercially available ELISA kit was employed (JEB-17789, Nanjing Jin Yibai Biological Technology Co. Ltd., China), and all procedures were carried out strictly according to the manufacturer's instructions. Briefly, standards with known concentrations of 1,5-AG and serum samples diluted 1:5 were added to 96-well microplates pre-coated with specific antibodies and incubated at 37°C for 60 minutes. After five washing cycles, the chromogenic substrate solution was added, and the plates were gently shaken to mix. Color development was performed at 37°C in the dark for 15 minutes, followed by the addition of 50 μL stop solution to each well to terminate the reaction. The optical density (OD) was then measured at 450 nm using a microplate reader.

Standard curves were generated for each batch by plotting blank-corrected OD values against the known concentrations of the calibrators and fitting a four-parameter logistic regression model. All standard curves demonstrated excellent goodness of fit, with R^2 values exceeding 0.999 for each batch.

Text S2. Instruments and Chemicals

Other instruments used in this study include a centrifuge (Heraeus Fresco17, Thermo Fisher Scientific, USA), a balance (BSA124S-CW, Sartorius, Germany), an ultrasonic homogenizer (PS-60AL, Shenzhen leaderbang, China), a tissue homogenizer(JXFSTPRP-24, Shanghai Jingxin Technology, China), and a freeze dryer (LGJ-10C, Foring Technology, China).

LC–MS grade methanol, acetonitrile and isopropanol were purchased from CNW Technologies (Shanghai, China). SIGMA-ALDRICH (St. Louis, MO, USA) supplied LC–MS-grade ammonium acetate and acetic acid. LC–MS grade ammonia was obtained from Fisher Chemical (Waltham, MA, USA). Ultrapure water was commercially sourced from Watsons (Hong Kong, China).

Text S3. UHPLC-HRMS Analytical Protocol

Prior to UHPLC-HRMS analysis, frozen serum samples stored at −80°C were thawed at 4°C. Aliquots (50 μL) were transferred to EP tubes, mixed with 200 μL of extraction solvent (methanol: acetonitrile = 1:1) containing isotope-labeled internal standard (IS), and vortexed for 30 seconds. The mixtures underwent ultrasonic extraction in an ice-water bath for 10 minutes, followed by incubation at −40°C for 1 hour. After centrifugation at 12,000 rpm (4°C, 15 minutes), the supernatants were subjected to UHPLC-HRMS analysis.

Supplementary Figures:

Figure S1. Representative EICs of the internal standard in QC samples. (A) positive ion mode; (B) negative ion mode. Note: EIC, extracted ion chromatogram; QC, quality control.

A

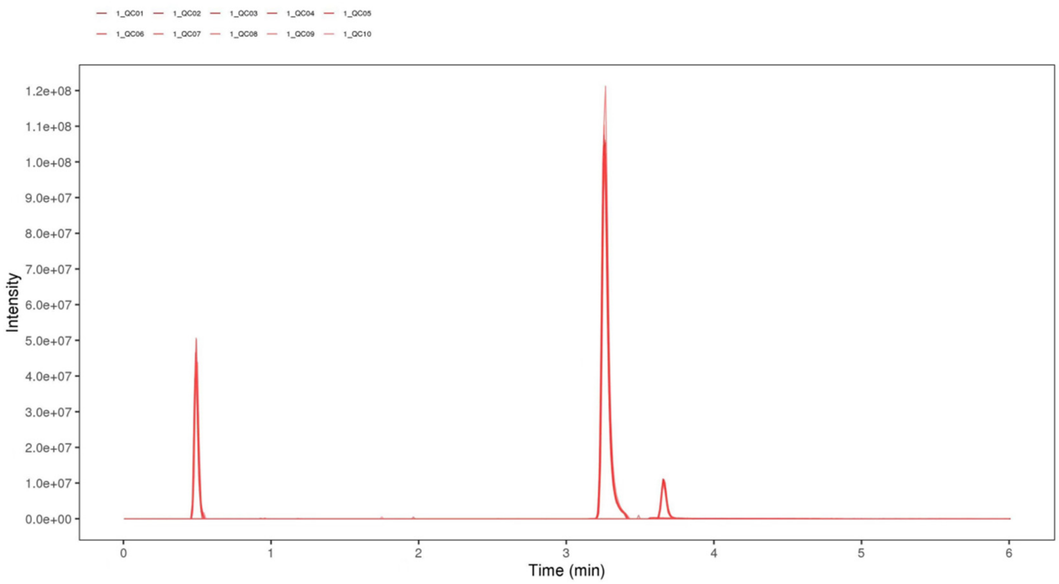

B

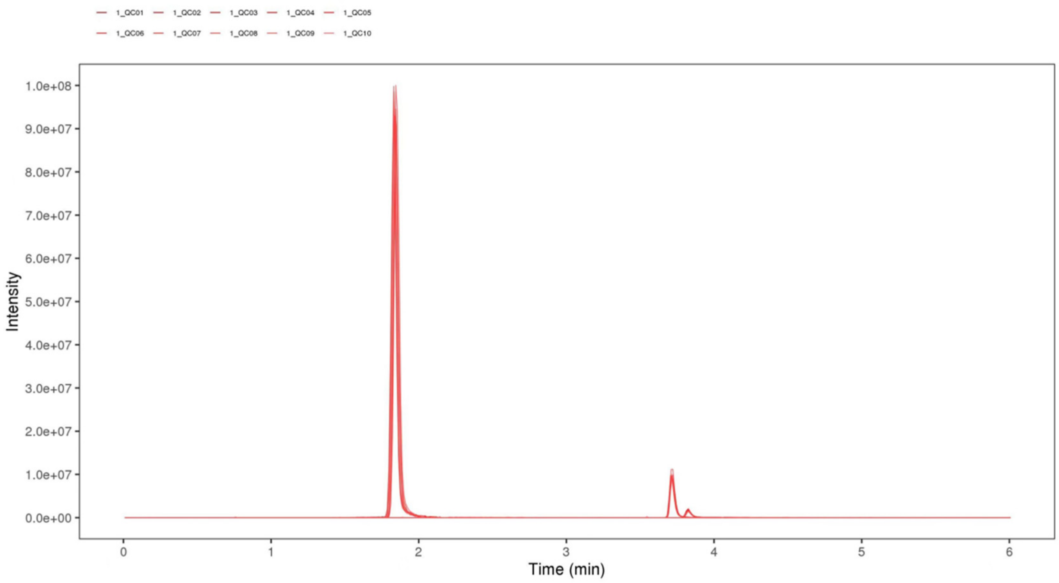

Figure S2. One-dimensional distribution plot of QC samples along the PCA-X axis. Note: QC, quality control; PCA-X, principal component analysis-x.

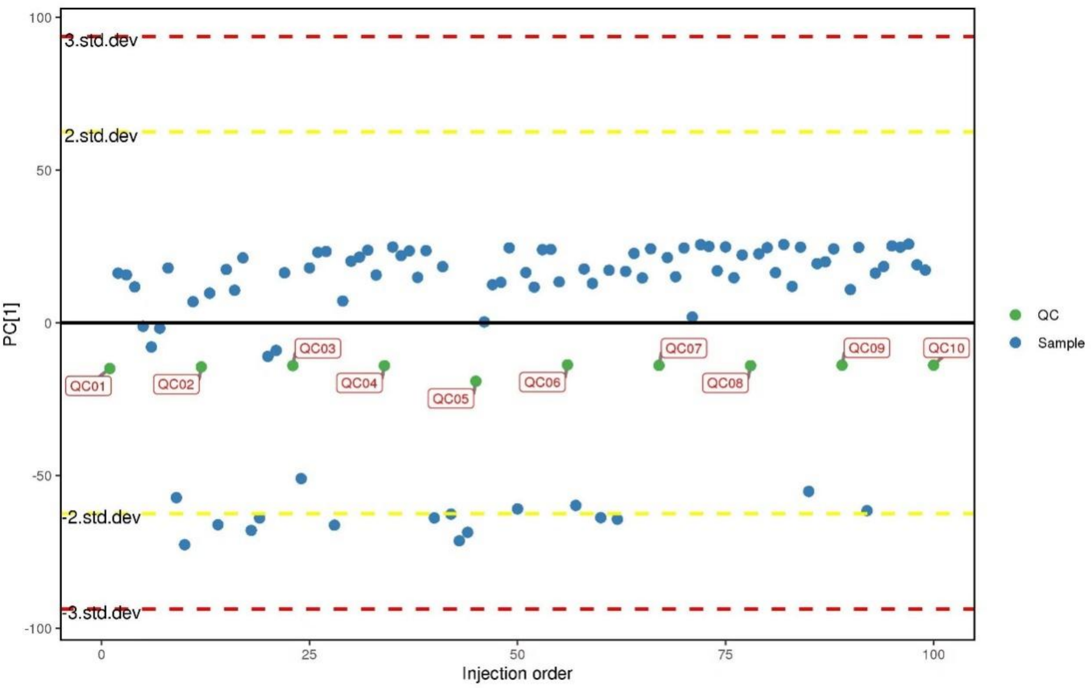

Figure S3. PCA score plots. (A) all three groups; (B) CON vs. PDM; (C) PDM vs. DM; (D) CON vs. DM. Note: PCA, principal component analysis; PDM, prediabetes; DM, diabetes mellitus; CON, control group.

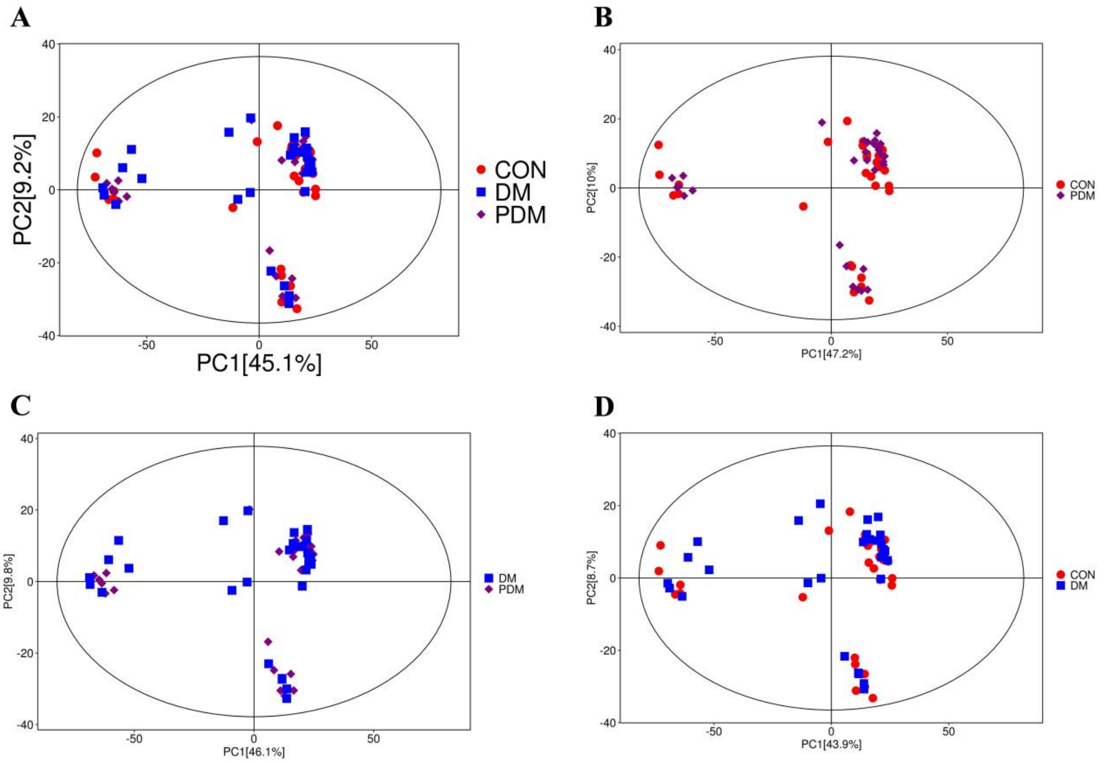

Figure S4. OPLS-DA score plots. (A) CON vs. PDM; (B) PDM vs. DM; (C) CON vs. DM. Note: OPLS-DA, orthogonal projection to latent structures-discriminant analysis; PDM, prediabetes; DM, diabetes mellitus; CON, control group.

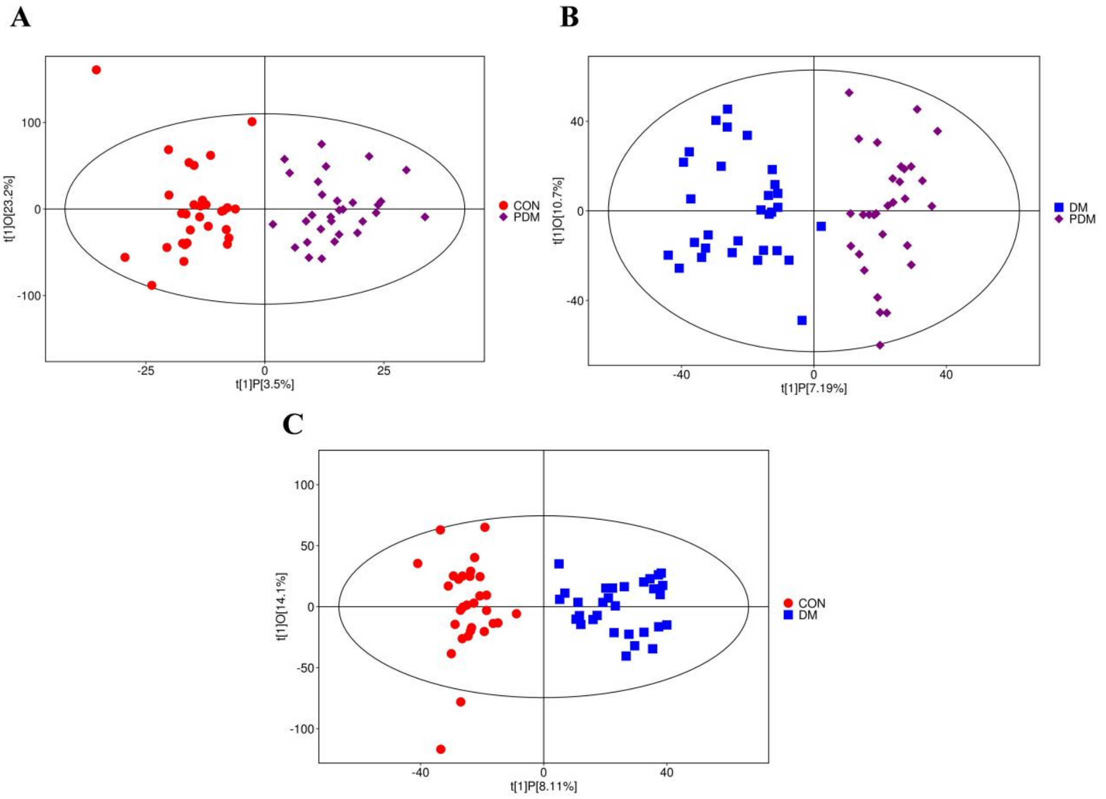

Figure S5. Permutation test results of OPLS-DA models. (A) CON vs. PDM; (B) PDM vs. DM; (C) CON vs. DM. Note: OPLS-DA, orthogonal projection to latent structures-discriminant analysis; PDM, prediabetes; DM, diabetes mellitus; CON, control group.

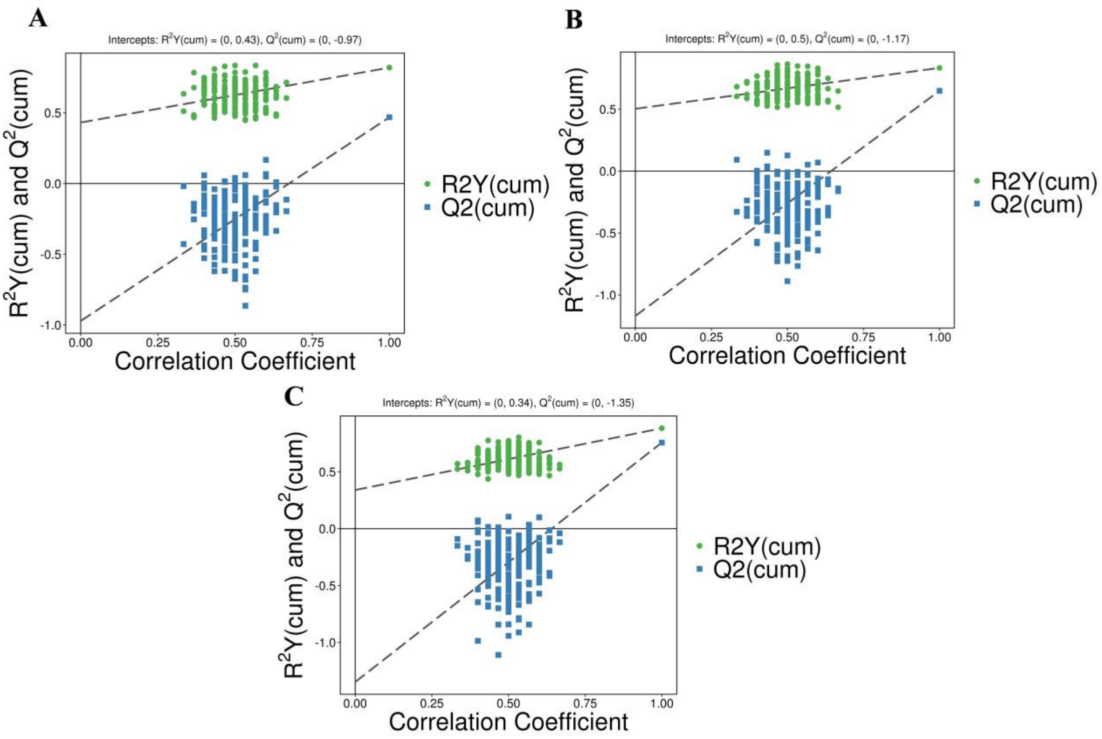

Figure S6. Venn diagram of differentially abundant metabolites among groups. Note: PDM, prediabetes; DM, diabetes mellitus; CON, control group.

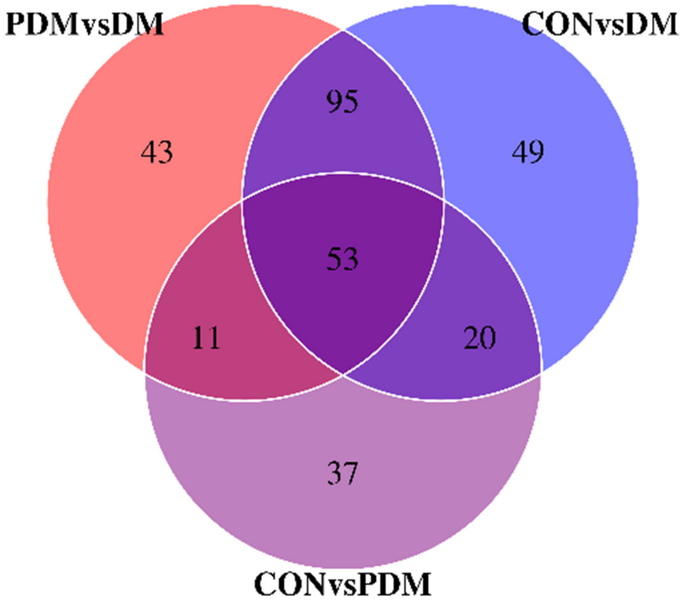

Figure S7. Bubble plots of metabolic pathway analysis. (A) CON vs. PDM; (B) PDM vs. DM; (C) CON vs. DM. Each bubble in the bubble plot represents a metabolic pathway. The x-axis position and bubble size reflect the pathway's impact value in topological analysis—the larger the bubble, the greater the impact. The y-axis position and color intensity indicate the significance of enrichment analysis (p-values are plotted as negative natural logarithm, i.e., −ln(p)); darker colors correspond to smaller p-values, signifying more significant enrichment. Note: PDM, prediabetes; DM, diabetes mellitus; CON, control group.

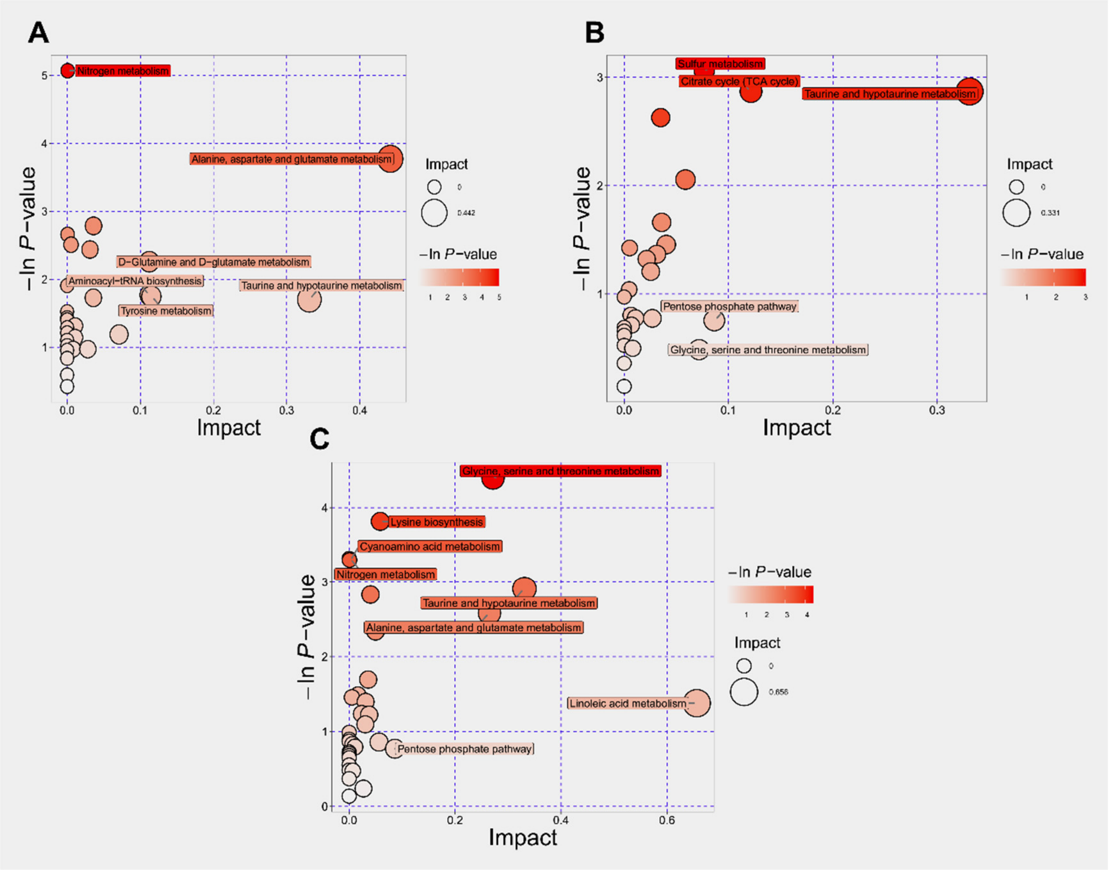

Figure S8. Comparison of serum 1,5-AG levels between groups at the 3-month endpoint. Error bars represent the standard error of the mean (SEM); baseline measurements were not available; therefore, the figure presents post-intervention absolute values for between-group comparison only. Note: 1,5-AG, 1,5-anhydroglucitol; D group, diet group; E group, exercise group; D+E group, diet + exercise group; C group, control group.

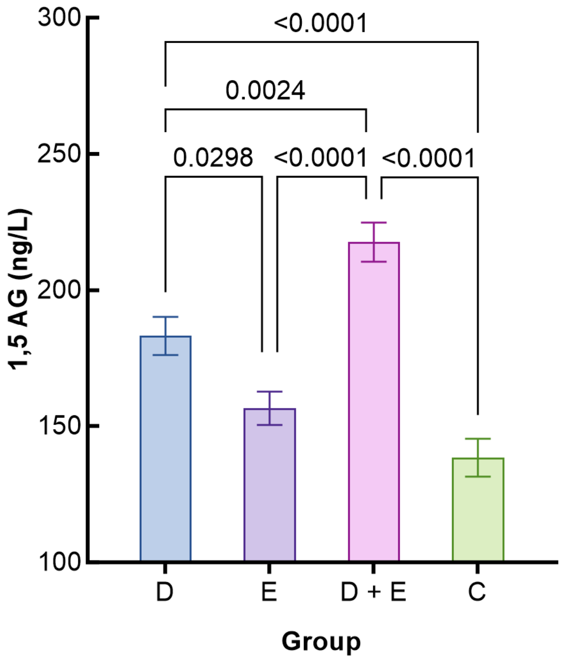

Supplementary Tables:

Table S1. CONSORT 2025 checklist item description.

Section/topic	No	CONSORT 2025 checklist item description	Reported on page no.
**Title and abstract**			
Title and structured abstract	1a	Identification as a randomised trial	1
	1b	Structured summary of the trial design, methods, results, and conclusions	1
**Open science**			
Trial registration	2	Name of trial registry, identifying number (with URL) and date of registration	8,9
Protocol and statistical analysis plan	3	Where the trial protocol and statistical analysis plan can be accessed	8,9,11
Data sharing	4	Where and how the individual de-identified participant data (including data dictionary), statistical code and any other materials can be accessed	12
Funding and conflicts of interest	5a	Sources of funding and other support (eg, supply of drugs), and role of funders in the design, conduct, analysis and reporting of the trial	12
	5b	Financial and other conflicts of interest of the manuscript authors	12
**Introduction**			
Background and rationale	6	Scientific background and rationale	2
Objectives	7	Specific objectives related to benefits and harms	2
**Methods**			
Patient and public involvement	8	Details of patient or public involvement in the design, conduct and reporting of the trial	8-10
Trial design	9	Description of trial design including type of trial (eg, parallel group, crossover), allocation ratio, and framework (eg, superiority, equivalence, non-inferiority, exploratory)	8-10
Changes to trial protocol	10	Important changes to the trial after it commenced including any outcomes or analyses that were not prespecified, with reason	11
Trial setting	11	Settings (eg, community, hospital) and locations (eg, countries, sites) where the trial was conducted	9
Eligibility criteria	12a	Eligibility criteria for participants	9
	12b	If applicable, eligibility criteria for sites and for individuals delivering the interventions (eg, surgeons, physiotherapists)	10
Intervention and comparator	13	Intervention and comparator with sufficient details to allow replication. If relevant, where additional materials describing the intervention and comparator (eg, intervention manual) can be accessed	10

Section/topic	No	CONSORT 2025 checklist item description	Reported on page no.
Outcomes	14	Prespecified primary and secondary outcomes, including the specific measurement variable (eg, systolic blood pressure), analysis metric (eg, change from baseline, final value, time to event), method of aggregation (eg, median, proportion), and time point for each outcome	11
Harms	15	How harms were defined and assessed (eg, systematically, non-systematically)	4
Sample size	16a	How sample size was determined, including all assumptions supporting the sample size calculation	9
	16b	Explanation of any interim analyses and stopping guidelines	4,9
Randomisation:			
Sequence generation	17a	Who generated the random allocation sequence and the method used	10
	17b	Type of randomisation and details of any restriction (eg, stratification, blocking and block size)	9
Allocation concealment mechanism	18	Mechanism used to implement the random allocation sequence (eg, central computer/telephone; sequentially numbered, opaque, sealed containers), describing any steps to conceal the sequence until interventions were assigned	9,10
Implementation	19	Whether the personnel who enrolled and those who assigned participants to the interventions had access to the random allocation sequence	9-11
Blinding	20a	Who was blinded after assignment to interventions (eg, participants, care providers, outcome assessors, data analysts)	9
	20b	If blinded, how blinding was achieved and description of the similarity of interventions	9
Statistical methods	21a	Statistical methods used to compare groups for primary and secondary outcomes, including harms	11
	21b	Definition of who is included in each analysis (eg, all randomised participants), and in which group	4
	21c	How missing data were handled in the analysis	4
	21d	Methods for any additional analyses (eg, subgroup and sensitivity analyses), distinguishing prespecified from post hoc	5,11
**Results**			
Participant flow, including flow diagram	22a	For each group, the numbers of participants who were randomly assigned, received intended intervention, and were analysed for the primary outcome	4
	22b	For each group, losses and exclusions after randomisation, together with reasons	4,9
Recruitment	23a	Dates defining the periods of recruitment and follow-up for outcomes of benefits and harms	4
	23b	If relevant, why the trial ended or was stopped	
Intervention and comparator delivery	24a	Intervention and comparator as they were actually administered (eg, where appropriate, who delivered the intervention/comparator, how participants adhered, whether they were delivered as intended (fidelity))	12
	24b	Concomitant care received during the trial for each group	10
Baseline data	25	A table showing baseline demographic and clinical characteristics for each group	4
Numbers analysed, outcomes and estimation	26	For each primary and secondary outcome, by group:<br>● the number of participants included in the analysis<br>● the number of participants with available data at the outcome time point<br>● result for each group, and the estimated effect size and its precision (such as 95% confidence interval)<br>● for binary outcomes, presentation of both absolute and relative effect size	5-7

Section/topic	No	CONSORT 2025 checklist item description	Reported on page no.
Harms	27	All harms or unintended events in each group	4
Ancillary analyses	28	Any other analyses performed, including subgroup and sensitivity analyses, distinguishing pre-specified from post hoc	5-7
**Discussion**			
Interpretation	29	Interpretation consistent with results, balancing benefits and harms, and considering other relevant evidence	3-8
Limitations	30	Trial limitations, addressing sources of potential bias, imprecision, generalisability, and, if relevant, multiplicity of analyses	8-9

Citation: Hopewell S, Chan AW, Collins GS, Hróbjartsson A, Moher D, Schulz KF, et al. CONSORT 2025 Statement: updated guideline for reporting randomised trials. BMJ. 2025; 389:e081123. https://dx.doi.org/10.1136/bmj-2024-081123
© 2025 Hopewell et al. This is an Open Access article distributed under the terms of the Creative Commons Attribution License (https://creativecommons.org/licenses/by/4.0/), which permits unrestricted use, distribution, and reproduction in any medium, provided the original work is properly cited.

*We strongly recommend reading this statement in conjunction with the CONSORT 2025 Explanation and Elaboration and/or the CONSORT 2025 Expanded Checklist for important clarifications on all the items. We also recommend reading relevant CONSORT extensions. See www.consort-spirit.org.

Table S2. Number of differential metabolites.

	Number of differential metabolites	Upregulated	Downregulated
CON vs PDM	121	101	20
PDM vs DM	202	66	136
CON vs DM	217	104	113

Table S3. Significantly differential metabolites between groups in serum samples.

No.	Metabolites	Formula	m/z	RT (s)	p A	q A, #	VIP A	AUC A	Validated AUC A	p B	q B, #	VIP B	AUC B	Validated AUC B	p C	q C, #	VIP C	AUC C	Validated AUC C	Change direction
1	Taurine	$C_2H_7NO_3S$	124.007	177.6	< 0.001	0.030	2.561	0.783 (0.660, 0.907)	0.663 (0.393, 0.932)	0.001	0.012	1.596	0.756 (0.632, 0.879)	0.563 (0.265, 0.860)	< 0.001	< 0.001	2.599	0.928 (0.857, 0.999)	0.900 (0.749, 1.000)	↑
2	Glucose	$C_6H_{12}O_6$	179.056	181.6	0.003	0.163	2.087	0.779 (0.654, 0.904)	0.750 (0.489, 1.000)	< 0.001	< 0.001	2.640	0.888 (0.800, 0.976)	0.775 (0.553, 0.997)	< 0.001	< 0.001	2.813	0.943 (0.884, 1.000)	0.950 (0.858, 1.000)	↑
3	1,5-AG	$C_6H_{12}O_5$	163.061	113.6	0.013	0.305	2.296	0.711 (0.573, 0.849)	0.738 (0.495, 0.980)	< 0.001	< 0.001	3.266	0.970 (0.928, 1.00)	0.938 (0.832, 1.000)	< 0.001	< 0.001	3.201	0.993 (0.982, 1.000)	1.000 (1.000, 1.000)	↓
4	Galactose	$C_6H_{12}O_6$	179.056	181.6	0.003	0.163	2.087	0.779 (0.654, 0.904)	0.750 (0.489, 1.000)	< 0.001	< 0.001	2.640	0.888 (0.800, 0.976)	0.775 (0.553, 0.997)	< 0.001	< 0.001	2.813	0.943 (0.884, 1.000)	0.950 (0.858, 1.000)	↑

No.	Metabolites	Formula	m/z	RT (s)	p [A]	q [A, #]	VIP [A]	AUC [A]	Validated AUC [A]	p [B]	q [B, #]	VIP [B]	AUC [B]	Validated AUC [B]	p [C]	q [C, #]	VIP [C]	AUC [C]	Validated AUC [C]	Change direction
5	Tagatose	$C_6H_{12}O_6$	203.052	180.2	0.001	0.059	2.407	0.798 (0.678, 0.917)	0.825 (0.618, 1.000)	< 0.001	< 0.001	2.737	0.892 (0.804, 0.981)	0.925 (0.804, 1.000)	< 0.001	< 0.001	2.941	0.957 (0.909, 1.000)	0.975 (0.916, 1.000)	↑
6	Phellopterin	$C_{17}H_{16}O_5$	339.060	179.6	0.001	0.094	2.126	0.749 (0.625, 0.873)	0.838 (0.637, 1.000)	< 0.001	< 0.001	2.856	0.898 (0.820, 0.976)	0.963 (0.889, 1.000)	< 0.001	< 0.001	3.033	0.974 (0.943, 1.000)	0.975 (0.916, 1.000)	↑
7	Sulfasalazine	$C_{18}H_{14}N_4O_5S$	399.080	179.6	< 0.001	0.007	2.621	0.803 (0.690, 0.917)	0.775 (0.540, 1.000)	< 0.001	< 0.001	2.670	0.881 (0.796, 0.967)	0.875 (0.712, 1.000)	< 0.001	< 0.001	3.048	0.977 (0.949, 1.000)	0.925 (0.792, 1.000)	↑
8	Imperatorin	$C_{16}H_{14}O_4$	269.088	177.3	0.003	0.147	2.413	0.761 (0.636, 0.886)	0.700 (0.443, 0.957)	< 0.001	< 0.001	1.656	0.781 (0.664, 0.898)	0.713 (0.432, 0.993)	< 0.001	< 0.001	2.469	0.921 (0.842, 1.000)	0.963 (0.881, 1.000)	↑

[#] value after false discovery rate (FDR)

Superscripts A, B and C indicate CON vs. PDM group, PDM vs. DM group and DM vs. CON group, respectively;

Note: CON, normoglycemic control group; PDM, prediabetes; DM, diabetes mellitus; RT, retention time; VIP, variable important in the projection; AUC, area under the curve.

Table S4. Baseline daily intake of food categories among RCT participants.

Variable	Total (n = 275)	D (n=72)	E (n=69)	D + E (n=66)	C (n=68)	*F*	*p*
Refined staple foods (g)	275.5 ± 103.5	268.5 ± 121.6	267.9 ± 108.9	275.1 ± 85.6	291.1 ± 93.0	0.748	0.524
Whole grains, starchy tubers, and pulses (g)	63.1 ± 45.8	69.9 ± 57.5	65.6 ± 41.2	53.9 ± 36.0	62.4 ± 44.3	1.514	0.211
Fried dough products (g)	3.8 ± 9.9	3.0 ± 8.8	3.2 ± 9.4	3.4 ± 10.5	5.5 ± 10.6	0.979	0.403
Soybeans and soy products (g)	23.9 ± 20.8	24.5 ± 23.2	24.7 ± 21.0	23.7 ± 18.5	22.7 ± 20.4	0.133	0.940
Vegetables (g)	351.3 ± 143.1	319.5 ± 140.0	372.8 ± 137.5	360.3 ± 165.1	354.2 ± 125.1	1.820	0.144
Fruits (g)	119.0 ± 113.8	109.6 ± 110.0	131.3 ± 120.1	114.0 ± 114.2	121.4 ± 112.2	0.481	0.696
Livestock meat (g)	56.2 ± 59.3	57.1 ± 56.2	51.7 ± 57.4	49.3 ± 54.1	66.4 ± 68.3	1.458	0.343
Poultry meat (g)	15.6 ± 25.6	14.8 ± 21.4	13.3 ± 23.3	16.3 ± 27.4	17.9 ± 30.2	1.117	0.749
Seafood (g)	27.3 ± 32.6	25.2 ± 26.3	31.7 ± 44.7	24.1 ± 28.5	28.0 ± 27.5	0.405	0.523
Dairy products (g)	81.4 ± 115.6	68.3 ± 94.1	102.9 ± 130.8	67.9 ± 103.1	86.7 ± 129.4	0.751	0.226
Eggs (g)	55.3 ± 39.8	50.4 ± 38.0	56.7 ± 40.7	53.0 ± 33.3	61.5 ± 46.1	1.012	0.388
Pastry and baked goods (g)	8.5 ± 14.8	9.0 ± 14.5	8.3 ± 13.8	7.0 ± 13.1	9.6 ± 17.5	0.383	0.766
Nuts and seeds (g)	13.2 ± 23.5	12.6 ± 23.1	9.0 ± 14.1	17.2 ± 28.5	14.0 ± 25.7	1.443	0.230

Quantitative data are presented as mean ± standard deviation (SD). Note: D group, diet group; E group, exercise group; D+E group, diet + exercise group; C group, control group.

Table S5. DM incidence in groups after 3-month intervention.

Variable	Total (n=275)	D (n=72)	E (n=69)	D+E (n=66)	C (n=68)	Statistic	*p*
Outcome, n (%)						χ^2=8.806	0.032
Non-DM	242 (88.00)	65 (90.28)	63 (91.30)	61 (92.42)	53 (77.94)		
DM	33 (12.00)	7 (9.72)	6 (8.70)	5 (7.58)	15 (22.06)		

Categorical data are represented by n (%).

Table S6. Daily dietary intake of participants before and 3-month after lifestyle intervention.

Variable	Baseline	3-month	*t*	*p*	Change	*F*	*p*
Dietary intake							
Energy (kJ)						3.835	**0.010**
D (n=72)	7537.7 ± 2180.6	6350.7 ± 1716.5 [a]	-4.504	< **0.001**	-1187.0 ± 2236.4 [a]		
E (n=69)	7616.2 ± 2190.4	7588.7 ± 1618.1 [b]	-0.095	0.924	-27.5 ± 2395.1 [b]		
D + E (n=66)	7520.4 ± 1825.7	6577.2 ± 1571.2 [a]	-3.471	< **0.001**	-943.2 ± 2207.7 [a,c]		
C (n=68)	8089.8 ± 2200.8	7809.1 ± 1524.7 [b]	-0.947	0.347	-280.7 ± 2444.3 [c]		
Energy (kcal)						3.835	**0.010**
D (n=72)	1801.6±521.2	1517.8 ± 410.3 [a]	-4.504	< **0.001**	-283.7 ± 534.5 [a]		
E (n=69)	1820.3±523.5	1813.7 ± 386.7 [b]	-0.095	0.924	-6.6 ± 572.5 [b]		
D + E (n=66)	1797.4±436.3	1572.0 ± 375.5 [a]	-3.471	< **0.001**	-225.4 ± 527.7 [a,c]		
C (n=68)	1933.5±526.0	1866.4 ± 364.4 [b]	-0.947	0.347	-67.1 ± 584.2 [c]		
Carbohydrates (g)						7.221	< **0.001**
D (n=72)	300.0±93.3	240.2±60.2 [a]	-5.865	< **0.001**	-59.8±86.5 [a]		
E (n=69)	304.0±88.3	296.8±60.5 [b]	-0.687	0.494	-7.2±87.4 [b,c]		
D + E (n=66)	296.2±68.0	239.1±58.6 [a]	-6.694	< **0.001**	-57.0±69.2 [a,]		
C (n=68)	315.5±75.8	298.9±52.6 [b]	-1.519	0.133	-16.6±90.2 [c]		
Fats (g)						0.353	0.787
D (n=72)	34.0±17.8	32.0±14.8	-0.787	0.434	-2.0±21.9		
E (n=69)	33.4±16.8	35.3±16.2	0.667	0.507	1.9±23.2		
D + E (n=66)	35.6±20.6	36.6±15.0	0.330	0.743	1.0±24.8		
C (n=68)	38.4±21.6	38.8±17.0	0.135	0.893	0.4±24.9		
Proteins (g)						0.136	0.938
D (n=72)	77.3±24.5	75.6±26.0	-0.454	0.651	-1.7±31.1		
E (n=69)	80.2±30.0	81.9±22.1	0.430	0.669	1.8±34.7		
D + E (n=66)	78.0±26.3	78.7±23.4	0.158	0.875	0.7±35.2		
C (n=68)	84.7±30.0	85.7±23.9	0.241	0.810	1.0±35.1		
Refined staple foods (g)						12.785	< **0.001**
D (n=72)	268.5 ± 121.6	174.6 ± 61.6 [a]	-6.349	< **0.001**	-93.9 ± 125.5 [a]		
E (n=69)	267.9 ± 108.9	259.4 ± 71.5 [b]	-0.681	0.498	-8.5 ± 103.7 [b,c]		
D + E (n=66)	275.1 ± 85.6	169.4 ± 83.8 [a,c]	-8.911	< **0.001**	-105.6 ± 96.3 [a]		
C (n=68)	291.1 ± 93.0	266.3 ± 71.1 [b,c]	-1.687	0.096	-24.8 ± 121.2 [c]		
Whole grains,						16.865	< **0.001**

Variable	Baseline	3-month	*t*	*p*	Change	*F*	*p*
starchy tubers, and pulses (g)							
D (n=72)	69.9 ± 57.5	112.4 ± 72.6 [a]	-3.749	**< 0.001**	42.5 ± 96.2 [a]		
E (n=69)	65.6 ± 41.2	65.4 ± 30.5 [b]	0.022	0.983	-0.1 ± 49.1 [b,c]		
D + E (n=66)	53.9 ± 36.0	116.9 ± 81.0 [a]	-6.029	**< 0.001**	63.2 ± 85.1 [a]		
C (n=68)	62.4 ± 44.3	60.3 ± 31.8 [b]	0.325	0.746	-2.1 ± 54.3 [c]		
Fried Dough Products (g)						0.047	0.986
D (n=72)	3.0 ± 8.8	2.9 ± 8.9	-0.064	0.949	-0.1 ± 12.5		
E (n=69)	3.2 ± 9.4	3.1 ± 7.6	-0.074	0.941	-0.1 ± 11.5		
D + E (n=66)	3.4 ± 10.5	3.0 ± 9.3	-0.227	0.821	-0.4 ± 12.9		
C (n=68)	5.5 ± 10.6	6.0 ± 12.3	0.238	0.812	0.5 ± 16.9		
Soybeans and Soy Products (g)						0.452	0.716
D (n=72)	24.5 ± 23.2	18.9 ± 12.6	-1.853	0.068	5.6 ± 25.5		
E (n=69)	24.7 ± 21.0	20.6 ± 14.4	-1.349	0.182	4.1 ± 25.4		
D + E (n=66)	23.7 ± 18.5	22.4 ± 17.5	-0.385	0.701	1.3 ± 26.6		
C (n=68)	22.7 ± 20.4	21.3 ± 16.6	-0.427	0.671	1.4 ± 27.2		
Vegetables (g)					不齐	7.785	**< 0.001**
D (n=72)	319.5 ± 140.0	478.9 ± 169.9 [a]	5.575	**< 0.001**	159.4 ± 242.7 [a]		
E (n=69)	372.8 ± 137.5	372.9 ± 143.9 [b]	0.005	0.996	0.1 ± 198.0 [b,c]		
D + E (n=66)	360.3 ± 165.1	414.0 ± 169.3 [b]	1.841	0.070	53.7 ± 236.8 [a,c]		
C (n=68)	354.2 ± 125.1	376.3 ± 117.3 [b]	1.111	0.271	22.1 ± 164.1 [b,c]		
Fruits (g)						0.599	0.616
D (n=72)	109.6 ± 110.0	113.4 ± 95.4	0.215	0.830	3.4 ± 16.5		
E (n=69)	131.2 ± 120.1	123.6 ± 104.0	-0.453	0.652	3.7 ± 16.3		
D + E (n=66)	114.0 ± 114.2	112.9 ± 103.1	-0.057	0.955	2.8 ± 15.8		
C (n=68)	121.4 ± 112.2	118.8 ± 100.4	-0.138	0.891	0.2 ± 18.4		
Livestock Meat (g)						0.069	0.976
D (n=72)	57.1 ± 56.2	69.9 ± 62.7	1.330	0.188	-3.8 ± 150.6		
E (n=69)	51.7 ± 57.4	59.6 ± 42.7	0.975	0.333	7.7 ± 141.0		
D + E (n=66)	49.3 ± 54.1	65.8 ± 51.2	1.778	0.080	1.1 ± 155.0		
C (n=68)	66.4 ± 68.3	69.9 ± 50.0	0.373	0.710	2.7 ± 158.7		
Poultry Meat (g)						0.337	0.799
D (n=72)	14.8 ± 21.4	18.0 ± 12.2	1.535	0.142	10.5 ± 131.8		
E (n=69)	13.3 ± 23.3	16.9 ± 34.3	0.698	0.487	-10.7 ± 171.0		
D + E (n=66)	16.3 ± 27.4	18.9 ± 16.1	1.340	0.188	10.7 ± 124.0		
C (n=68)	17.9 ± 30.2	12.0 ± 27.0	-1.136	0.260	8.8 ± 161.3		
Seafood (g)						0.384	0.765
D (n=72)	25.2 ± 26.3	33.4 ± 31.4	1.872	0.065	12.8 ± 81.5		
E (n=69)	31.7 ± 44.7	31.0 ± 27.0	-0.118	0.906	7.9 ± 67.2		
D + E (n=66)	24.1 ± 28.5	35.6 ± 40.6	1.873	0.066	16.6 ± 75.7		

Variable	Baseline	3-month	*t*	*p*	Change	*F*	*p*
C (n=68)	28.0 ± 27.5	30.5 ± 28.5	0.483	0.631	3.5 ± 76.9		
Dairy Products (g)						1.009	0.389
D (n=72)	68.3 ± 94.1	78.8 ± 105.4	0.673	0.503	8.2 ± 37.2		
E (n=69)	102.9 ± 130.8	92.216 ± 120.151	-0.519	0.605	-0.7 ± 51.7		
D + E (n=66)	67.9 ± 103.0	78.6 ± 101.9	0.704	0.484	11.6 ± 50.2		
C (n=68)	86.7 ± 129.4	95.4 ± 102.2	0.448	0.655	2.5 ± 41.8		
Eggs (g)						0.644	0.587
D (n=72)	50.4 ± 38.0	52.5 ± 21.1	0.391	0.697	2.1 ± 45.4		
E (n=69)	56.7 ± 40.7	46.4 ± 34.5	-1.991	0.050	-10.3 ± 43.0		
D + E (n=66)	53.0 ± 33.3	49.5 ± 24.0	-0.741	0.461	-3.5 ± 38.7		
C (n=68)	61.5 ± 46.1	58.6 ± 63.3	-0.311	0.756	-2.9 ± 76.9		
Pastry and Baked Goods (g)						0.690	0.559
D (n=72)	9.0 ± 14.5	4.7 ± 14.8	-1.774	0.080	-4.2 ± 20.2		
E (n=69)	8.3 ± 13.7	9.4 ± 16.6	0.440	0.661	1.1 ± 21.0		
D + E (n=66)	6.9 ± 13.1	6.5 ± 19.2	-0.163	0.871	-0.5 ± 24.0		
C (n=68)	9.6 ± 17.5	8.2 ± 17.3	-0.447	0.656	-1.4 ± 25.2		
Nuts and Seeds (g)						0.851	0.467
D (n=72)	12.6 ± 23.1	11.6 ± 17.1	-0.302	0.763	-1.0 ± 27.9		
E (n=69)	9.0 ± 14.1	16.1 ± 20.1	2.372	**0.021**	7.1 ± 24.9		
D + E (n=66)	17.3 ± 28.5	18.1 ± 20.3	0.210	0.834	0.9 ± 35.2		
C (n=68)	14.0 ± 25.7	17.1 ± 25.7	0.682	0.497	3.0 ± 36.8		

Quantitative data are presented as mean ± standard deviation (SD). Different lowercase letters indicate statistically significant differences between groups (Dunnett's test for equal variances or Dunnett's T3 test for unequal variances, $P < 0.05$). Note: D group, diet group; E group, exercise group; D+E group, diet + exercise group; C group, control group.
