# Supplementary figures and images for: Effects of Balanced Dietary Patterns and/or Integrated Exercise on Serum 1,5-Anhydroglucitol and CVD Risk Factors in Individuals with Prediabetes
Source: Life (Basel). 2026 Jan 25;16(2):198. doi: 10.3390/life16020198 (PMC12941816; doi:10.3390/life16020198)

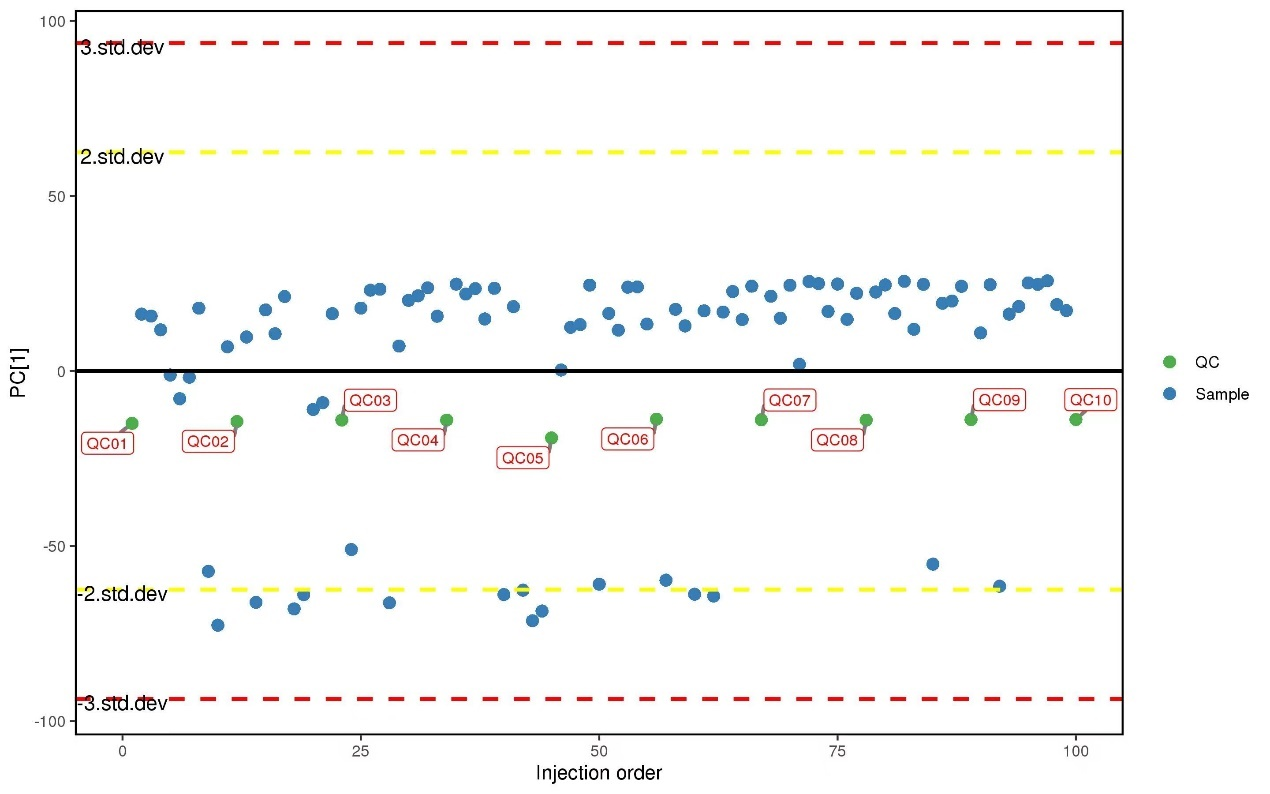

Supplement: Supplementary file 1 [file life-16-00198-s001.zip › Figure S2.tif]

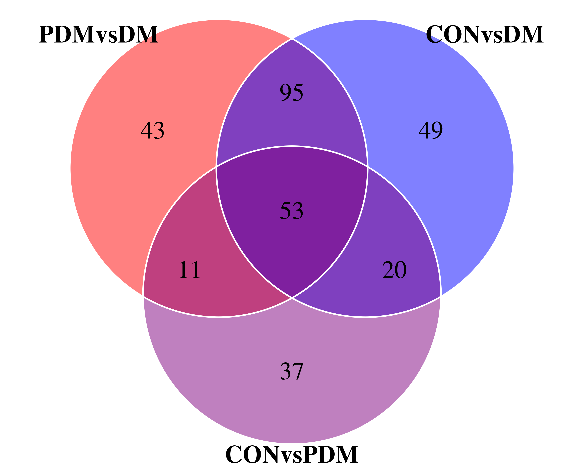

Supplement: Supplementary file 1 [file life-16-00198-s001.zip › Figure S6.tif]

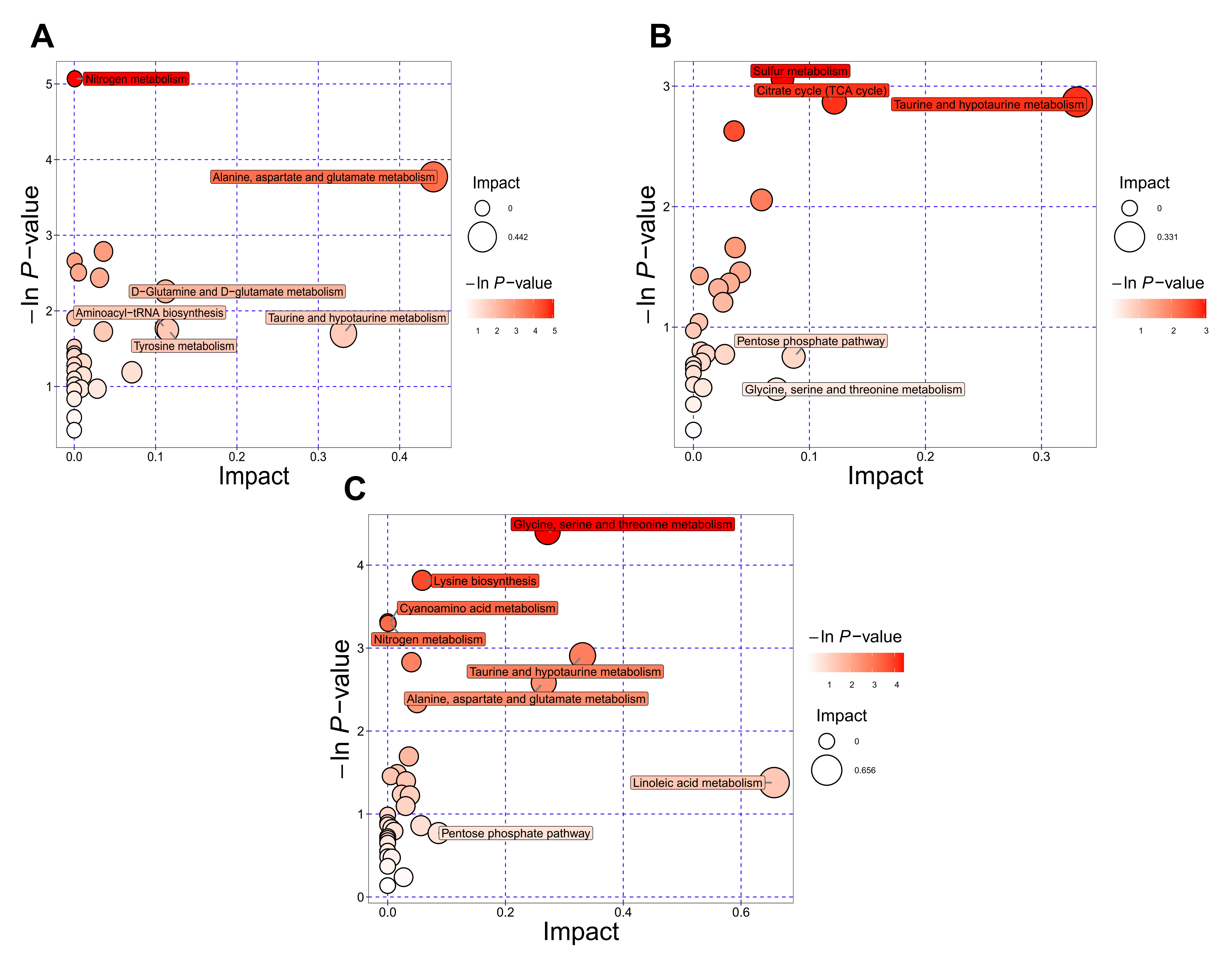

Supplement: Supplementary file 1 [file life-16-00198-s001.zip › Figure S7.tif]

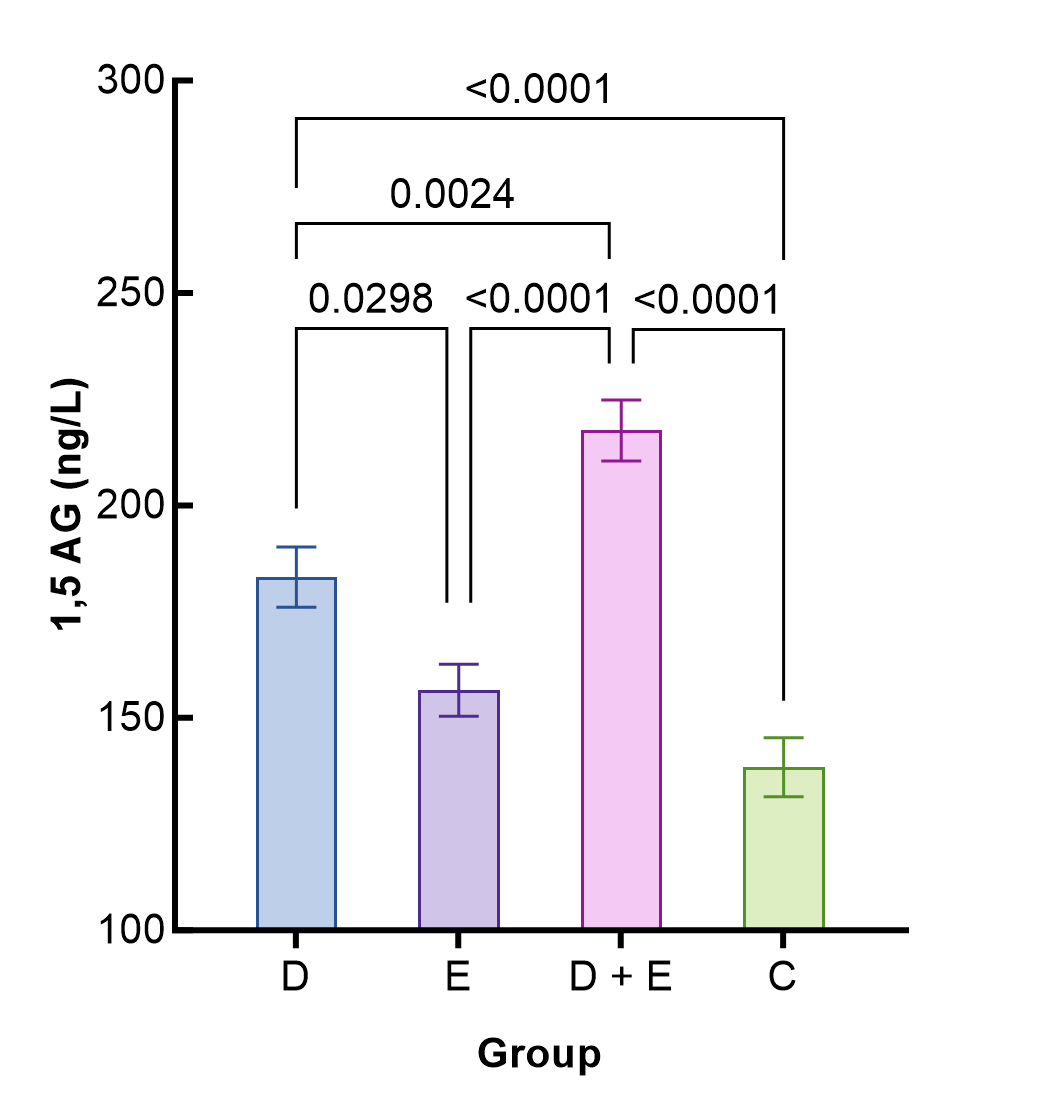

Supplement: Supplementary file 1 [file life-16-00198-s001.zip › Figure S8.tif]
